# Supplementary material for: Systemic miR-26a deficiency attenuates pulmonary fibrosis via PTEN upregulation and downstream TIMP-1 suppression
Source: Mol Ther Nucleic Acids. 2025 Nov 5;36(4):102765. doi: 10.1016/j.omtn.2025.102765 (PMC12663618; doi:10.1016/j.omtn.2025.102765)
Supplement: Document S1. Figures S1–S7 [file mmc1.pdf]

OMTN, Volume *36*

Supplemental information

Systemic miR-26a deficiency attenuates pulmonary fibrosis via PTEN upregulation and downstream TIMP-1 suppression

Arisa Hamada, Kiyofumi Shimoji, Taku Nakashima, Kakuhiro Yamaguchi, Shinjiro Sakamoto, Yasushi Horimasu, Takeshi Masuda, Hiroshi Iwamoto, Hironobu Hamada, Yun Guo, Tomoharu Yasuda, Shigeru Miyaki, and Noboru Hattori

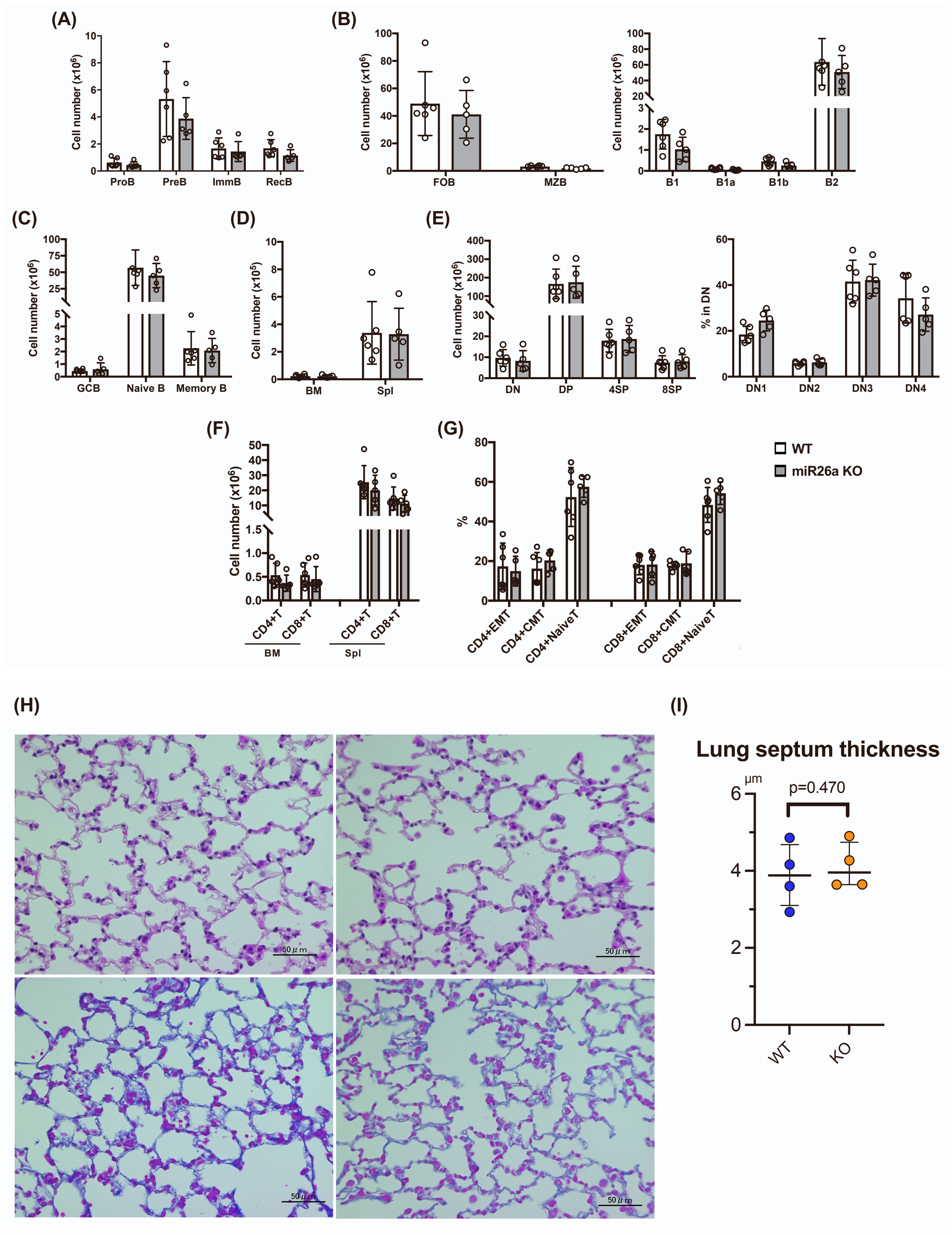
(A)
Cell number (x10^6)
ProB
PreB
ImmB
RecB
(B)
Cell number (x10^6)
FOB
MZB
Cell number (x10^6)
B1
B1a
B1b
B2
(C)
Cell number (x10^6)
GCB
Naive B
Memory B
(D)
Cell number (x10^5)
BM
Spl
(E)
Cell number (x10^6)
DN
DP
4SP
8SP
% in DN
DN1
DN2
DN3
DN4
(F)
Cell number (x10^6)
CD4+T
CD8+T
CD4+T
CD8+T
BM
Spl
(G)
%
CD4+EMT
CD4+CMT
CD4+NaiveT
CD8+EMT
CD8+CMT
CD8+NaiveT
WT
miR26a KO
(H)
50 μm
50 μm
50 μm
50 μm
(I)
Lung septum thickness
μm
p=0.470
WT
KO

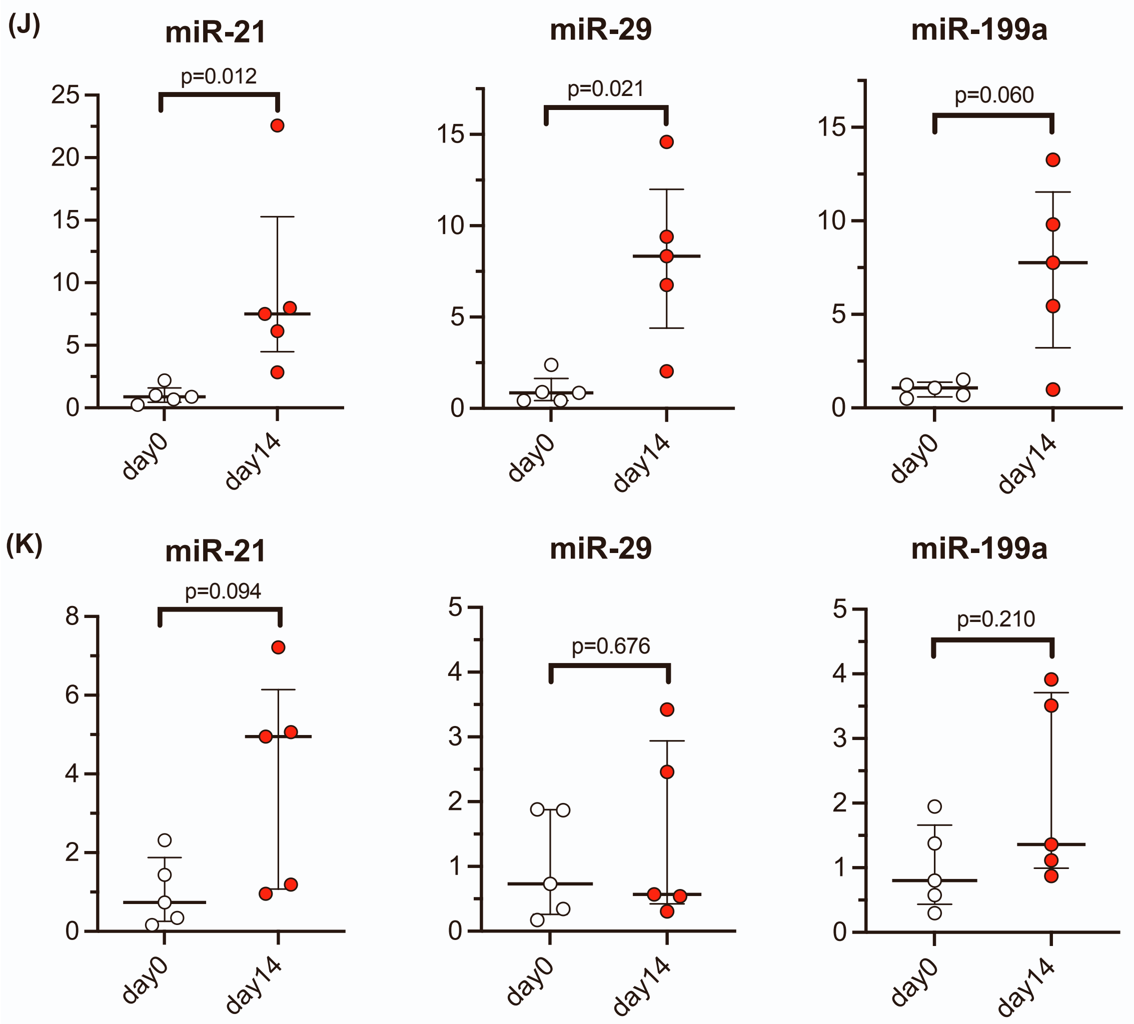

Figure S1

(A–G) Flow cytometry analysis of immune cells in bone marrow (BM), spleen (Spl), and thymus obtained from WT (*white*) and miR-26a KO (*grey*) mice (n = 5–6/group). (A) B cell development in BM. Pro-B cells, B220^{+}CD19^{-}; Pre-B cells, B220^{+}CD19^{+}IgM^{-}IgD^{-}; Immature (Imm) B cells, B220^{+}CD19^{+}IgM^{+}IgD^{low}; and Recirculating (Rec) B cells, B220^{+}CD19^{+}IgM^{+}IgD^{high}. (B and C) B1 and B2 cell development in spleen. (B) Follicular (FO) B cells, CD19^{+}B220^{+}AA4.1^{-}CD21^{low}CD23^{high}; Marginal zone (MZ) B cells, CD19^{+}B220^{+}AA4.1^{-}CD21^{high}CD23^{low}; B1 cells, CD19^{+}B220^{low}; B1a cells, CD19^{+}B220^{low}CD5^{+}IgM^{high}; B1b cells, CD19^{+}B220^{low}CD5^{-}IgM^{high}; and B2 cells, CD19^{+}B220^{+} corresponding to FO and MZ B cells. (C) Naive B cells, B220^{+}CD19^{+}CD38^{+}Fas^{-}; Germinal center (GC) B cells, B220^{+}CD19^{+}CD38^{-}Fas^{+}; and Memory B cells, B220^{+}CD19^{+}CD38^{+}CD73^{+}. (D) CD138^{+}TACI^{+} plasma cells in BM and spleen. (E) T cell development in thymus. DN, CD4^{-}CD8^{-}; DP, CD4^{+}CD8^{+}; 4SP,

$CD4^+CD8^-$; and 8SP, $CD4^-CD8^+$. DN cells were further fractioned to DN1 to DN4. DN1, $CD25^-CD44^+$; DN2, $CD25^+CD44^+$; DN3, $CD25^+CD44^-$; and DN4, $CD25^-CD44^-$. (F) Peripheral T cells in BM and spleen. $CD4^+$ T cells, $CD4^+CD8^-$; and $CD8^+$ T cells, $CD4^-CD8^+$. (G) Effector and memory development of $CD4^+$ and $CD8^+$ T cells in spleen. Naive T cells, $CD62L^+CD44^-$; Effector memory T cells (EMT), $CD62L^-CD44^+$; and Central memory T cells (CMT), $CD62L^+CD44^+$.
(H) Hematoxylin and eosin (HE) staining and Azan staining of WT and miR-26a KO murine lungs. Scale bars: 50 µm.
(I) The equivalent diameter of the lung septum thickness was measured and images were quantified using ImageJ2 (version 2.16.0/1.54p) (n = 4/group).
(J, K) miR-21, miR-29, and miR-199a levels in the lungs of (J) WT and (K) miR-26a KO mice before and on 14 days after BLM administration (n = 5/group).

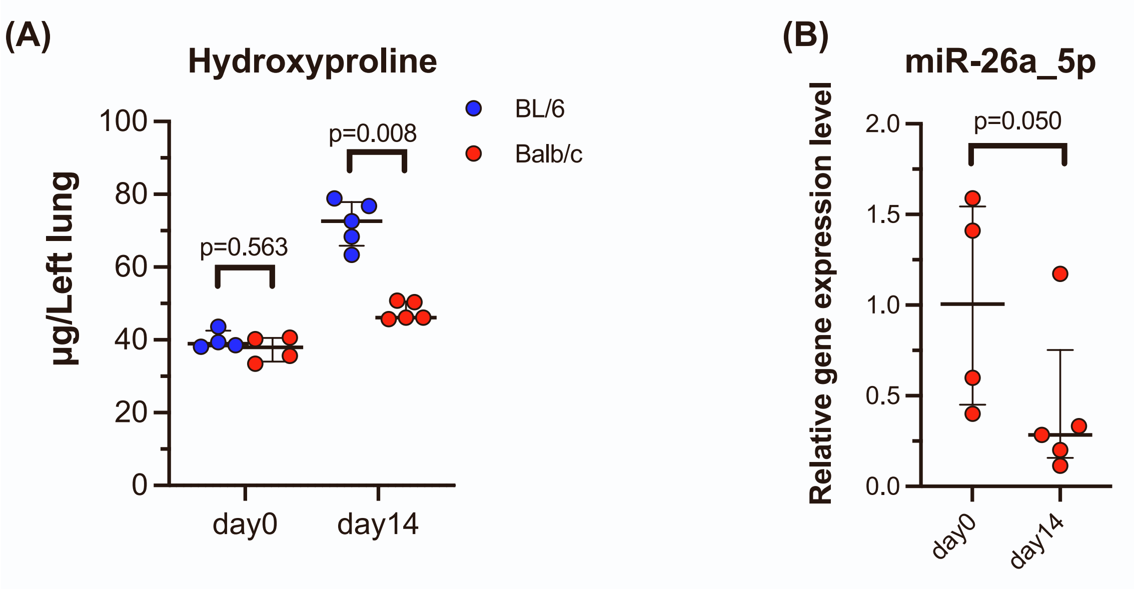

Figure S2

(A) Hydroxyproline levels in the left lung of C57BL/6J and BALB/cJ mice were measured 14 days after BLM administration (n = 4–5/group).

(B) miR-26a levels in the lungs of C57BL/6J and BALB/cJ mice before and on 14 days after BLM administration (n = 4–5/group).

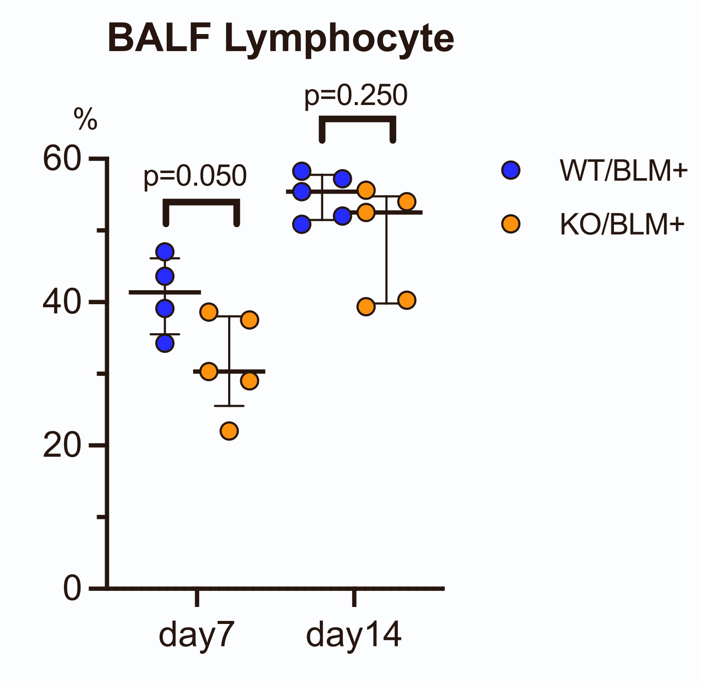

Figure S3

Lymphocytes percentages in BALF were measured 7 and 14 days after BLM administration (n = 4–5/ group).

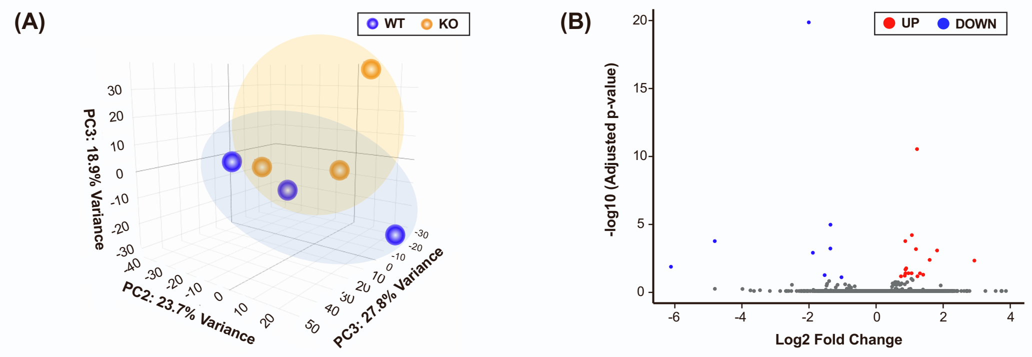

(C)

UP / DOWN	-log10 (FDR)	Fold enriched	Pathway
Upregulated	4.02	78.715	Circadian rhythm
Upregulated	1.64	24.441	Metabolism of xenobiotics by cytochrome P450
Downregulated	1.32	50.688	ECM-receptor interaction
Downregulated	1.32	44.605	Toll-like receptor signaling pathway
Downregulated	1.32	70.802	GnRH secretion
Downregulated	1.32	41.301	Protein digestion and absorption
Downregulated	1.32	32.798	Apelin signaling pathway

Figure S4

RNA sequencing of WT and miR-26a KO murine lungs was conducted on before BLM administration (n = 3/group).

(A) Each dot represents one sample subjected to principal component analysis (PCA).

(B) Volcano plot of differential gene expression between WT and miR-26a KO groups (n = 3/group) : the upregulated genes are dotted in red and downregulated in blue.

(C) Top seven enriched signaling pathways following KEGG enrichment analysis.

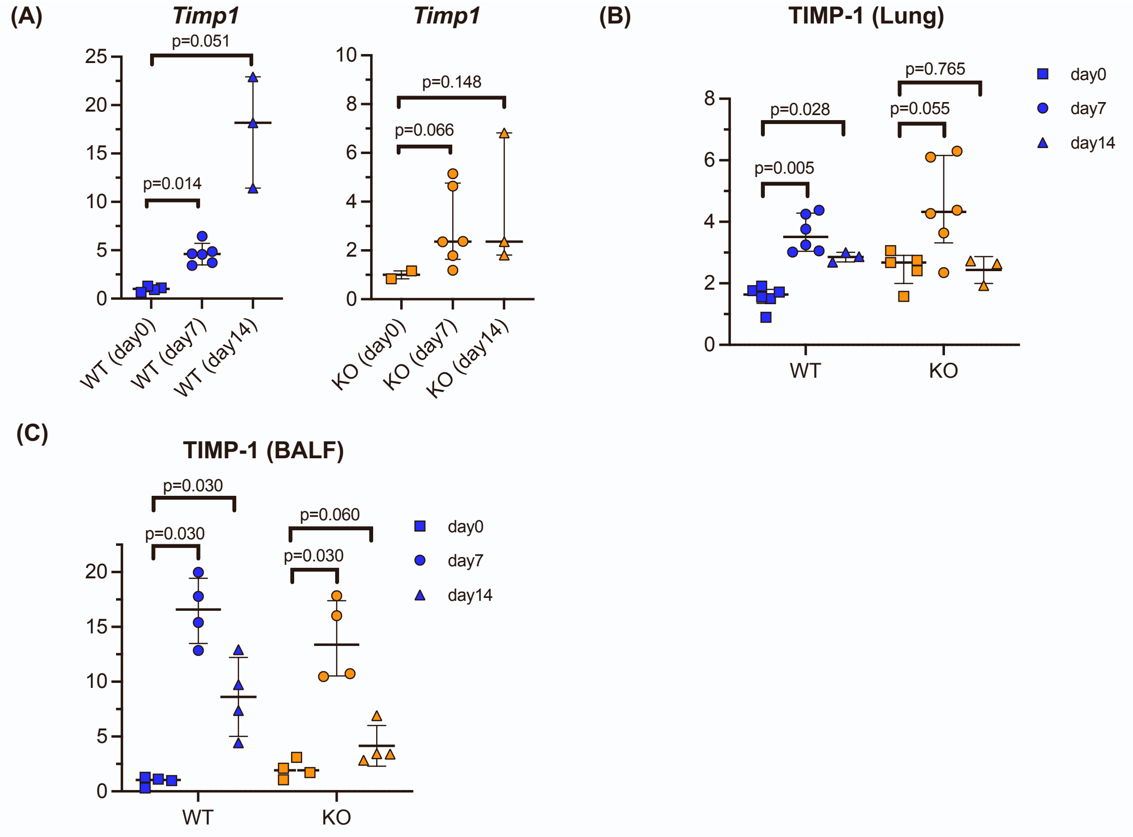

Figure S5

(A) Quantitative PCR analysis of *Timp1* mRNA expression in murine lungs before and on 7 and 14 days after BLM administration (n = 2–6/group).

(B, C) ELISA analysis of TIMP-1 protein levels in murine lungs (ng/mg Protein) and BALF (ng/mL) before and on 7 and 14 days after BLM administration (n = 3–6/group).

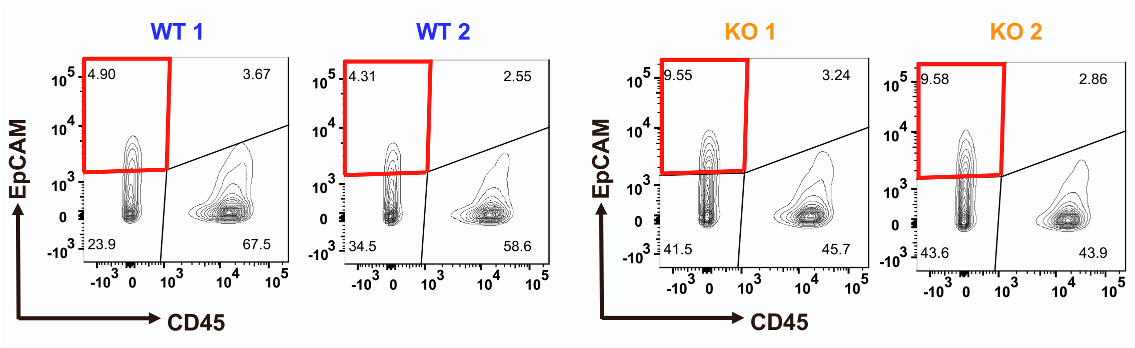

Figure S6

Flow cytometry analysis of the percentage of epithelial cells (defined as $CD45^{-}/EpCAM^{+}$) in total lung cells on 7 days after BLM administration in WT and miR-26a KO mice.

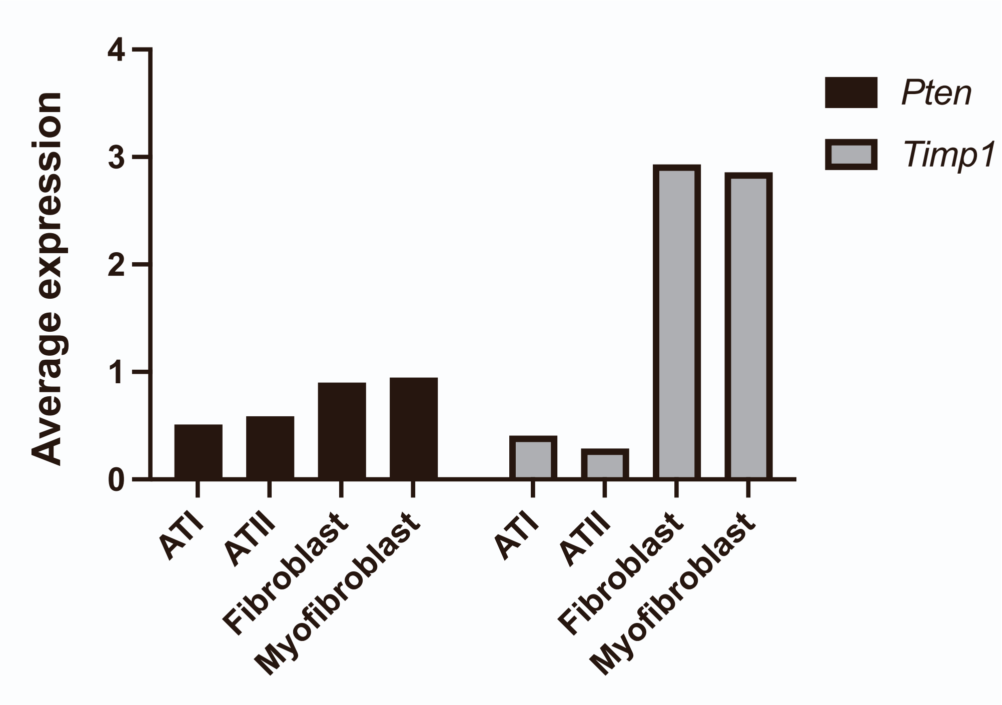

Figure S7

Supplemental figures were created with reference to the publicly available results presented on the IPF Cell Atlas website (http://ipfcellatlas.com/). No additional analyses were performed, and the original data are derived from single-cell transcriptomic data of human lung cells from patients with idiopathic pulmonary fibrosis (IPF) reported by Adams et al. (Nat Med 2020). Average expression represents the log_{1+x}-transformed normalized expression values (log-normalized UMI counts) as shown in the IPF Cell Atlas.
