# Supplementary material for: Systemic miR-26a deficiency attenuates pulmonary fibrosis via PTEN upregulation and downstream TIMP-1 suppression
Source: Mol Ther Nucleic Acids. 2025 Nov 5;36(4):102765. doi: 10.1016/j.omtn.2025.102765 (PMC12663618; doi:10.1016/j.omtn.2025.102765)
Supplement: Document S2. Article plus supplemental information [file mmc2.pdf]

Molecular Therapy
Nucleic Acids
Original Article

Systemic miR-26a deficiency attenuates pulmonary fibrosis via PTEN upregulation and downstream TIMP-1 suppression

Arisa Hamada,[1,7] Kiyofumi Shimoji,[1,7] Taku Nakashima,[1] Kakuhiro Yamaguchi,[1] Shinjiro Sakamoto,[1] Yasushi Horimasu,[1] Takeshi Masuda,[1] Hiroshi Iwamoto,[1] Hironobu Hamada,[2] Yun Guo,[3] Tomoharu Yasuda,[3] Shigeru Miyaki,[4,5,6] and Noboru Hattori[1]

[1]Department of Molecular and Internal Medicine, Graduate School of Biomedical and Health Sciences, Hiroshima University, Hiroshima, Japan; [2]Department of Physical Analysis and Therapeutic Sciences, Graduate School of Biomedical and Health Sciences, Hiroshima University, Hiroshima, Japan; [3]Department of Immunology, Graduate School of Biomedical and Health Sciences, Hiroshima University, Hiroshima, Japan; [4]Department of Orthopedic Surgery, Graduate School of Biomedical and Health Sciences, Hiroshima University, Hiroshima, Japan; [5]Medical Center for Translational and Clinical Research, Hiroshima University Hospital, Hiroshima, Japan; [6]Department of Histology and Molecular Cell Biology, Faculty of Medicine / Graduate School of Medicine, Kagawa University, Kagawa, Japan

Several microRNAs (miRNAs) have been implicated in the pathophysiology of pulmonary fibrosis; however, the detailed mechanisms remain unclear. miR-26a has demonstrated antifibrotic effects, particularly when its expression is suppressed in the airways. However, the effects of systemic miR-26a deficiency on pulmonary fibrosis have not been investigated. We found that miR-26a knockout (KO) mice exhibited reduced pulmonary fibrosis compared with wild-type (WT) mice. Whole-lung RNA sequencing analysis indicated that the mammalian target of rapamycin complex 1 (MTORC1) signaling and phosphoinositide 3-kinase/protein kinase B (PI3K/AKT) signaling pathways were elevated in the WT group compared with the KO group. Loss of miR-26a increases *PTEN* expression, a target gene of miR-26a, resulting in the reduction of *Timp1* levels downstream of the PI3K/Akt-mTOR pathway, thereby attenuating fibrosis. Transfection with miR-26a significantly suppressed *Pten* expression and increased *Timp1* and *Acta2* expression in primary lung fibroblasts *in vitro*. These results are consistent with the *in vivo* findings, which suggest that miR-26a promotes fibrosis, contrary to previous reports indicating an antifibrotic role for miR-26a. Our findings suggest that local and systemic inhibition of miR-26a may exert opposing effects, highlighting the importance of careful interpretation of miR-26a-targeted therapeutic strategies.

INTRODUCTION

Idiopathic pulmonary fibrosis (IPF) is a disease characterized by the repeated damage and repair of alveolar epithelial cells (AECs), leading to the destruction of alveolar structures and subsequent fibrosis. IPF is an intractable disease without definitive treatment, and the average survival time after diagnosis is 3–5 years. Although several microRNAs (miRNAs) have been implicated in the pathogenesis of IPF, the detailed mechanisms remain unclear.[1] miRNAs are single-stranded non-coding RNAs, typically 20–25 bases long, that bind to the 3′ untranslated region (3′ UTR) of target messenger RNAs with complementary sequences and play a role in regulating gene expression. Recently, miRNAs have been reported to be involved in processes such as development, differentiation, and cell proliferation and have been associated with various diseases, including pulmonary fibrosis.[2,3]

Among these miRNAs, miR-26a has been shown to suppress the expression of mothers against decapentaplegic homolog 4 (Smad4) and connective tissue growth factor (CTGF) and has been reported to have protective effects against lung fibrosis.[4] Liang et al.[5] have reported that miR-26a expression was decreased in the lungs of mice with bleomycin (BLM)-induced pulmonary fibrosis compared with healthy mice, as well as in the lungs of patients with IPF compared with healthy controls. Furthermore, the administration of antagomiR-26a, an miR-26a antagonist, into the trachea of mice reduced miR-26a expression in the lungs, worsened fibrosis, and increased hydroxyproline levels in the lung tissue. Conversely, intratracheal administration of agomiR-26a, an miR-26a agonist, resulted in reduced fibrosis and lower hydroxyproline levels in the lung tissue after BLM administration. The underlying mechanism appears to be that miR-26a acts in a lung-protective manner by suppressing CTGF, which promotes collagen production and fibrosis. Additionally, BLM stimulation increases transforming growth factor β (TGF-β) levels, which further stimulates Smad3 phosphorylation and nuclear translocation, resulting in decreased miR-26a expression. This decrease in miR-26a levels increases CTGF levels, thereby promoting collagen production and exacerbating fibrosis.

Received 5 February 2025; accepted 31 October 2025;
https://doi.org/10.1016/j.omtn.2025.102765.

[7]These authors contributed equally

Correspondence: Taku Nakashima, MD, PhD, Department of Molecular and Internal Medicine, Graduate School of Biomedical and Health Sciences, Hiroshima University, 1-2-3 Kasumi, Minami-ku, Hiroshima 734-8551, Japan.
E-mail: tnaka@hiroshima-u.ac.jp

© 2025 The Author(s). Published by Elsevier Inc. on behalf of The American Society of Gene and Cell Therapy. This is an open access article under the CC BY license (http://creativecommons.org/licenses/by/4.0/).

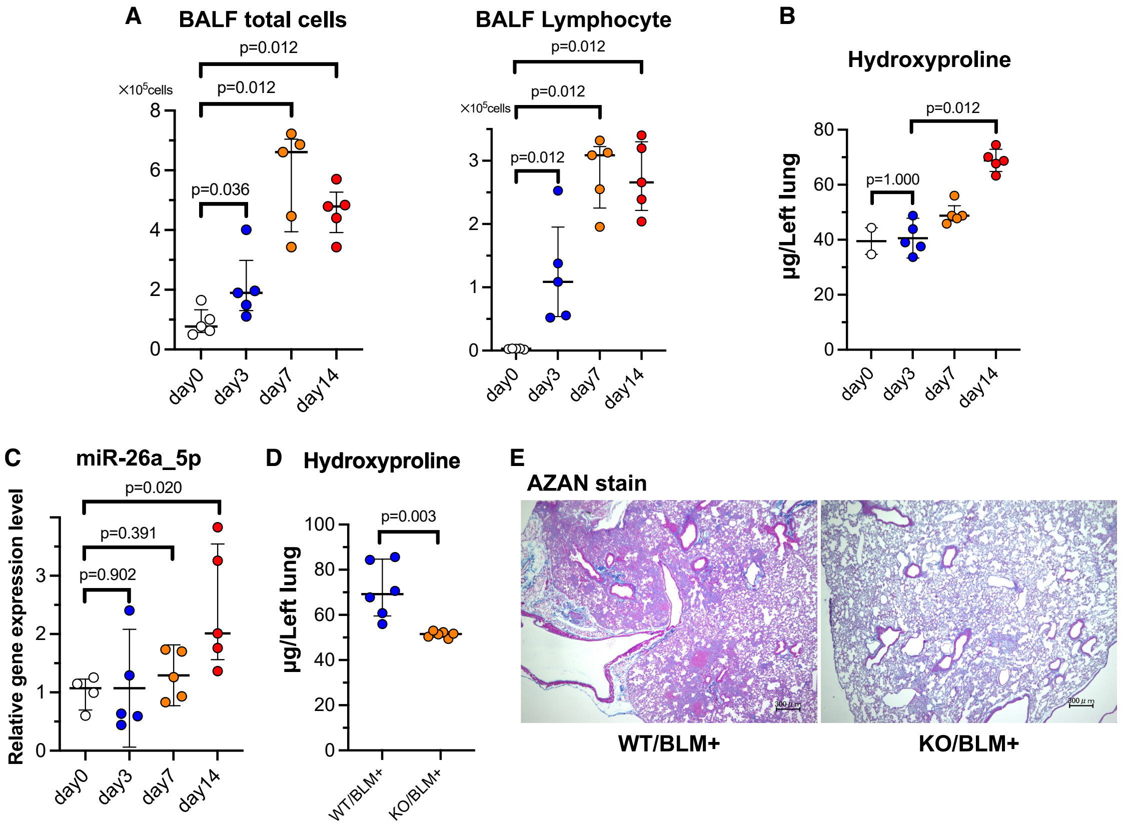

Figure 1. Evaluation of pulmonary fibrosis induced by BLM in miR-26a KO mice

(A) Total cell and lymphocyte counts in BALF were measured 14 days after BLM administration (n = 5/group). (B) Hydroxyproline levels in the left murine lung were measured 14 days after BLM administration (n = 2–5/group). (C) Time course of miR-26a expression in BLM-injured murine lungs measured using quantitative PCR (n = 4–5/group). (D) Hydroxyproline levels in the left murine lung were measured 14 days after BLM administration (n = 6/group). (E) Representative images of Azan staining (high-power field) of murine lung sections. Scale bars, 300 μm.

While miR-26a has been reported to have antifibrotic effects, these effects have primarily been observed with the suppression of miR-26a localized to the airways. However, the effects of systemic miR-26a deficiency on pulmonary fibrosis have not yet been investigated. Therefore, we conducted a study to determine whether systemic suppression of miR-26a would similarly exacerbate pulmonary fibrosis, as observed with airway-limited suppression of miR-26a, by administering BLM to miR-26a knockout (KO) mice and comparing the results with those from a wild-type (WT) control group.

RESULTS

Evaluation of pulmonary fibrosis induced by BLM in miR-26a KO mice

First, we performed flow cytometry using the bone marrow, thymus, spleen, mesenteric lymph nodes, and peripheral blood leukocytes to analyze the immune cells in miR-26a KO mice. The results showed no significant abnormalities in the differentiation, maintenance, or distribution of B cells and T cells and no significant abnormalities in the production of memory B and T cells (Figures S1A–S1G). Furthermore, no significant difference in alveolar septal thickness was observed between the lung tissue sections of 8-week-old WT and KO mice, as analyzed using hematoxylin and eosin (H&E) staining and Azan staining (Figures S1H and S1I).

Next, we investigated the dynamics of miR-26a in C57BL/6J WT mice after BLM oropharyngeal aspiration (OA). Total cell and lymphocyte counts in the bronchoalveolar lavage fluid (BALF) were significantly increased by BLM OA (Figure 1A), and hydroxyproline levels were significantly elevated on day 14 compared with those on days 0 and day 3 (Figure 1B), confirming the induction of pulmonary fibrosis by BLM OA. miR-26a expression in the lungs, as measured using quantitative polymerase chain reaction (PCR), was significantly higher on day 14 than on day 0 (Figure 1C). We also measured the levels of other fibrosis-related miRNAs (miR-21, miR-29, and miR-199a) in the lungs of C57BL/6J WT and miR-26a KO mice and observed significant upregulations of fibrosis-related miRNAs 14 days after BLM OA in WT (Figure S1J) but not in KO mice (Figure S1K). Furthermore, in BALB/cJ mice, which are resistant to fibrosis, the hydroxyproline levels on day 14 after BLM OA were significantly lower than those in C57BL/6J mice

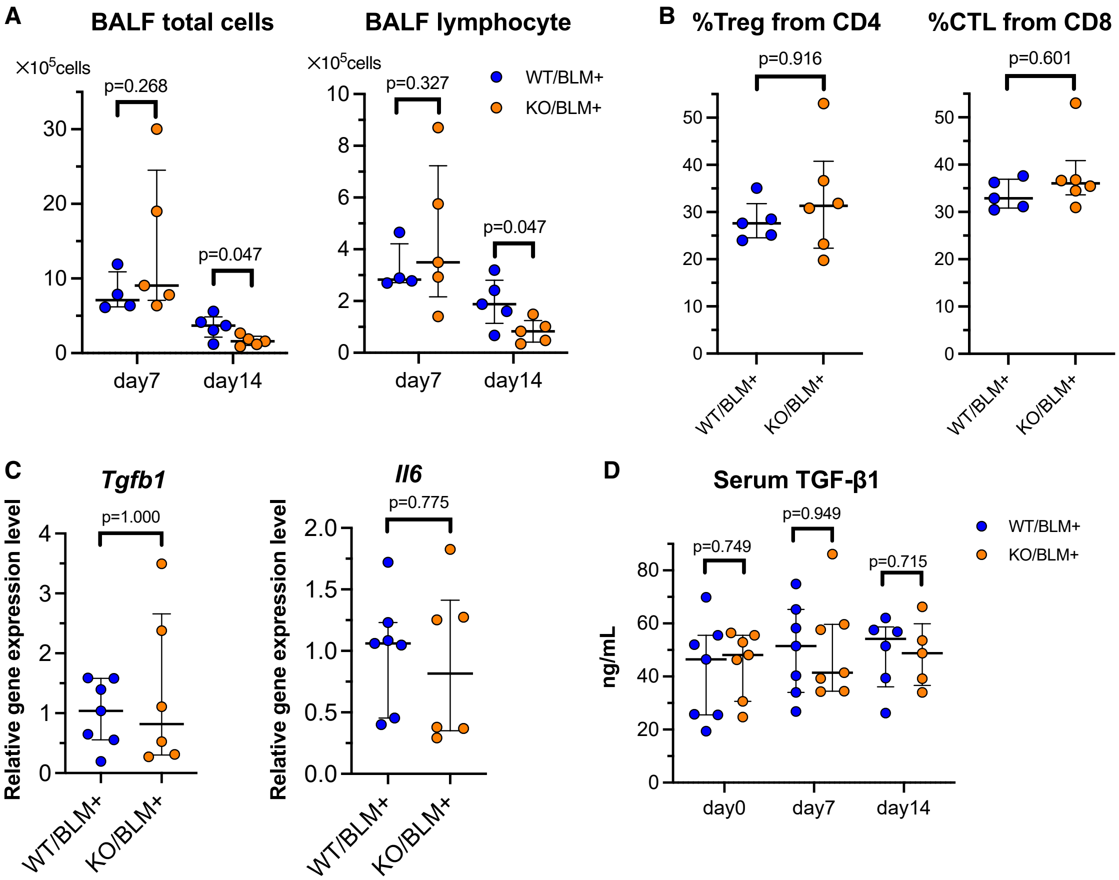

Figure 2. Cytokine evaluation in miR-26a KO mice

(A) Total cell and lymphocyte counts in BALF were measured 7 and 14 days after BLM administration (n = 4–5/group). (B) Flow cytometry analysis of regulatory T cells (Tregs; CD3+/CD4+/CD25+) within CD4+ T cells and cytotoxic T lymphocytes (CTLs; CD3+/CD8+) within CD3+ T cells using murine lung 14 days after BLM administration (n = 5–6/group). (C) Quantitative PCR analysis of *Tgfb1* and *Il6* mRNA expression levels in murine lungs 14 days after BLM administration (n = 6–7/group). (D) ELISA analysis of TGF-β1 protein levels in the serum before and on 7 and 14 days after BLM administration (n = 5–7/group).

(Figure S2A). Unlike in C57BL/6J mice, no significant increase in pulmonary miR-26a expression was observed in BALB/cJ mice (Figure S2B).

We then compared the effects of BLM OA between miR-26a KO and WT mice (littermates without mutations were used). The hydroxyproline levels on day 14 were significantly lower in the KO group than in the WT group (Figure 1D). Histological analyses using Azan staining of lung tissues showed that fibrosis was less severe in miR-26a KO mice than in WT mice, which was consistent with the observed reduction in the hydroxyproline levels (Figure 1E).

Cytokine evaluation in miR-26a KO mice

To investigate the potential reasons for the milder pulmonary fibrosis observed in miR-26a KO mice than in WT mice, we evaluated inflammation-related factors in the lungs and blood. In BALF analysis on days 7 and 14 after BLM OA, the total cell and lymphocyte counts were significantly lower in the KO group on day 14 (Figure 2A; lymphocyte [%] data are shown in Figure S3). Flow cytometry analysis of lung tissue 14 days after BLM OA revealed no significant differences between WT and miR-26a KO mice in the proportions of regulatory T cells (Tregs, defined as CD3+/CD4+/CD25+) within CD4+ T cells and cytotoxic T lymphocytes (CTLs, defined as CD3+/CD8+) within CD3+ T cells (Figure 2B).

Regarding the mRNA expression levels of *Tgfb1 and Il6* in the lung tissue on day 14 after BLM OA, no significant differences were observed between WT and miR-26a KO mice (Figure 2C). Similarly, serum TGF-β1 levels on days 0, 7, and 14 after BLM OA also showed no significant differences between the two groups (Figure 2D).

Suppression of the fibrosis-promoting molecule TIMP-1 via suppressing PI3K/Akt pathway in miR-26a KO mice

No significant differences in inflammatory factors were observed between the WT and miR-26a KO mice. To identify the factors that suppressed fibrosis in the KO mice, we performed a comprehensive

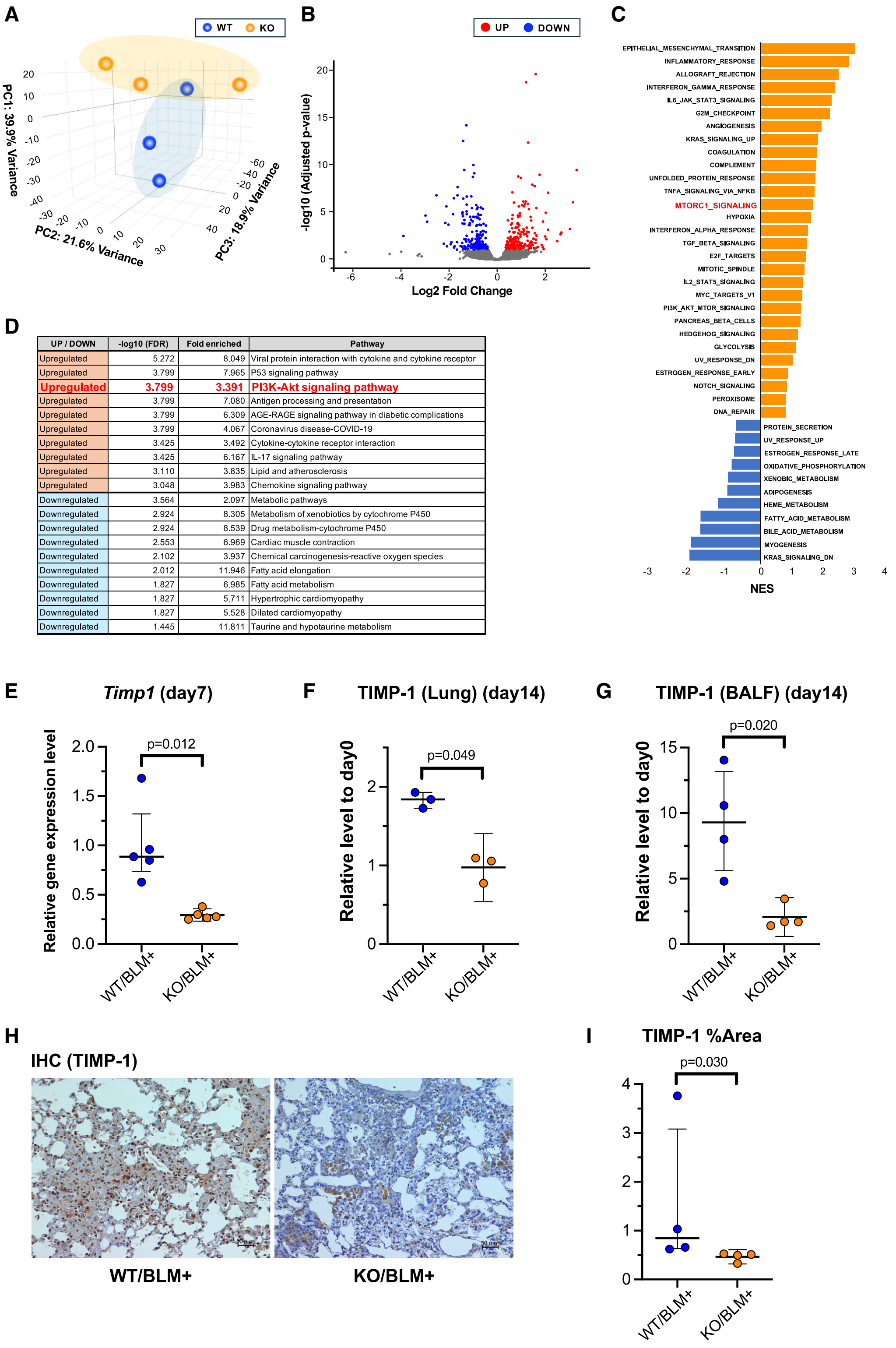

UP / DOWN	-log10 (FDR)	Fold enriched	Pathway
Upregulated	5.272	8.049	Viral protein interaction with cytokine and cytokine receptor
Upregulated	3.799	7.965	P53 signaling pathway
**Upregulated**	**3.799**	**3.391**	**PI3K-Akt signaling pathway**
Upregulated	3.799	7.080	Antigen processing and presentation
Upregulated	3.799	6.309	AGE-RAGE signaling pathway in diabetic complications
Upregulated	3.799	4.067	Coronavirus disease-COVID-19
Upregulated	3.425	3.492	Cytokine-cytokine receptor interaction
Upregulated	3.425	6.167	IL-17 signaling pathway
Upregulated	3.110	3.835	Lipid and atherosclerosis
Upregulated	3.048	3.983	Chemokine signaling pathway
Downregulated	3.564	2.097	Metabolic pathways
Downregulated	2.924	8.305	Metabolism of xenobiotics by cytochrome P450
Downregulated	2.924	8.539	Drug metabolism-cytochrome P450
Downregulated	2.553	6.969	Cardiac muscle contraction
Downregulated	2.102	3.937	Chemical carcinogenesis-reactive oxygen species
Downregulated	2.012	11.946	Fatty acid elongation
Downregulated	1.827	6.985	Fatty acid metabolism
Downregulated	1.827	5.711	Hypertrophic cardiomyopathy
Downregulated	1.827	5.528	Dilated cardiomyopathy
Downregulated	1.445	11.811	Taurine and hypotaurine metabolism

(legend on next page)

comparison of total lung mRNA expression levels between the WT and KO groups using RNA sequencing analysis. Principal-component analysis (PCA) revealed no clear difference in distribution, and Kyoto Encyclopedia of Genes and Genomes (KEGG) enrichment analysis showed no significant pathways associated with pulmonary fibrosis between the WT and KO groups on day 0 (Figures S4A–S4C). PCA showed that the WT and KO groups on day 7 were clearly distinct, indicating a difference in gene expression between the groups (Figures 3A and 3B). Hallmark analysis revealed that MTORC1 signaling was elevated in the WT group compared with that in the KO group (Figure 3C). Additionally, KEGG enrichment analysis showed that the PI3K/AKT signaling pathway was enriched in the WT group compared with the KO group (Figure 3D).

To further investigate this, we focused on individual genes related to fibrosis and those downstream of the PI3K/Akt-mTOR pathway. *Timp1* mRNA expression in the lung tissue of the KO group showed a marked decrease on day 7 after BLM OA compared with that in the WT group (Figures 3E and S5A). At the protein level, enzyme-linked immunosorbent assay (ELISA) measurements showed that lung TIMP-1 levels on day 14 were significantly suppressed in the KO group compared with those in the WT group (Figures 3F and S5B). Similarly, the increase in BALF TIMP-1 on day 14 was also suppressed in the KO group compared with that in the WT group (Figures 3G and S5C). Immunostaining of lung tissue samples collected on day 14 after BLM administration further confirmed that TIMP-1 expression was reduced in the KO group compared with the WT group (Figure 3H), which was also statistically validated through quantitative analysis using ImageJ2 (Figure 3I).

PTEN as a direct target of miR-26a

We further explored genes as regulators of the PI3K/AKT pathway that could potentially be targets of miR-26a. This analysis led us to focus on the *Pten* gene, which has been shown to be a target of miR-26a (TargetScan: http://www.targetscan.org/) (Figure 4A). Using lung tissue samples collected on day 7 after BLM administration, we measured *Pten* mRNA levels and found that *Pten* expression was significantly elevated in the KO group compared with that in the WT group (Figure 4B). Additionally, PTEN protein levels were quantified using ELISA, showing that the KO group had significantly higher PTEN protein levels in the lung tissue on day 14 than the WT group (Figure 4C). Immunostaining further confirmed that PTEN expression was enhanced in the KO group compared with the WT group (Figure 4D), which was also statistically validated through quantitative analysis (Figure 4E). In flow cytometry analysis of total lung cells on day 7 after BLM OA, the percentage of CD45−/EpCAM+ cells, defined as epithelial cells, was approximately 9.5% in the KO/BLM group, compared with less than 5% in the WT/BLM group (Figure S6).

Differences in the effects of miR-26a by cell type

To explore the potential cell-type-specific effects of miR-26a, which might explain the reduced pulmonary fibrosis observed in miR-26a KO mice, we transfected miR-26a into the fibroblasts derived from the whole lungs of WT and KO mice and measured the mRNA expression levels of fibrosis-related genes using quantitative PCR. Transfection with miR-26a significantly increased miR-26a levels in both groups, confirming successful transfection (Figures 5A and 5E). Regarding *Pten* expression, transfection with miR-26a significantly suppressed *Pten* in both groups (Figures 5B and 5F). Furthermore, *Timp1* and *Acta2* expression was significantly increased in both groups after miR-26a transfection (Figures 5C, 5D, 5G, and 5H). A similar difference was observed in the LA-4 epithelial cells transfected with miR-26a, which showed suppressed *Pten* expression and increased *Timp1* and *Acta2* expression (Figures 5J, 5K, and 5L). Consistently, the human lung fibroblast cell line MRC-5 and the human alveolar epithelial cell line A549 transfected with miR-26a exhibited similar expression changes, showing decreased *Pten* and increased *Timp1* and *Acta2* expression (Figures 6A–6H).

Systemic miR-26a KO enhances PTEN expression and suppresses the fibrosis-promoting molecule TIMP-1 by inhibiting the PI3K/Akt-mTOR pathway, thereby attenuating pulmonary fibrosis, as summarized in graphical abstract.

DISCUSSION

In this study, we demonstrated for the first time that miR-26a KO mice exhibited milder pulmonary fibrosis than WT mice following intratracheal BLM administration. A previous study conducted at different time points has reported decreased miR-26a expression in the lungs of BLM-injured mice and patients with IPF.[5] Additionally, the intratracheal administration of antagomiR-26a, an miR-26a antagonist, in mice reduced miR-26a expression in the lungs and exacerbated pulmonary fibrosis. Conversely, intratracheal administration of agomiR-26a, an miR-26a agonist, ameliorated histological fibrosis and hydroxyproline levels in the lung tissue following BLM administration. Based on this report, we hypothesized that miR-26a KO mice would exhibit exacerbated pulmonary fibrosis compared with WT mice following BLM administration. However,

Figure 3. Suppression of the fibrosis-promoting molecule TIMP-1 via inhibition of the PI3K/Akt pathway in miR-26a KO mice
RNA sequencing of WT and miR-26a KO murine lungs was conducted on day 7 after BLM administration (n = 3/group). (A) Each dot represents one sample subjected to principal-component analysis (PCA). (B) Volcano plot of differential gene expression between WT and miR-26a KO groups (n = 3/group): the upregulated genes are dotted in red and downregulated in blue. (C) Upregulated and downregulated terms in the hallmark analysis when comparing the WT and miR-26a KO group. (D) Top 10 enriched signaling pathways following KEGG enrichment analysis. (E) Quantitative PCR analysis of *Timp1* mRNA expression in murine lungs on day 7 after BLM administration (n = 5/group). (F and G) ELISA analysis of TIMP-1 protein levels in murine lungs and BALF 14 days after BLM administration (n = 3–4/group). Values were normalized to the mean value of the day 0 control. (H) Immunohistochemical staining of WT and miR-26a KO murine lungs on day 14 after BLM administration using anti-TIMP-1 antibodies. Scale bars, 50 μm. (I) Quantification of the stained area (% of the total field) in the immunohistochemical staining images shown in Figure 3H (n = 4/group).

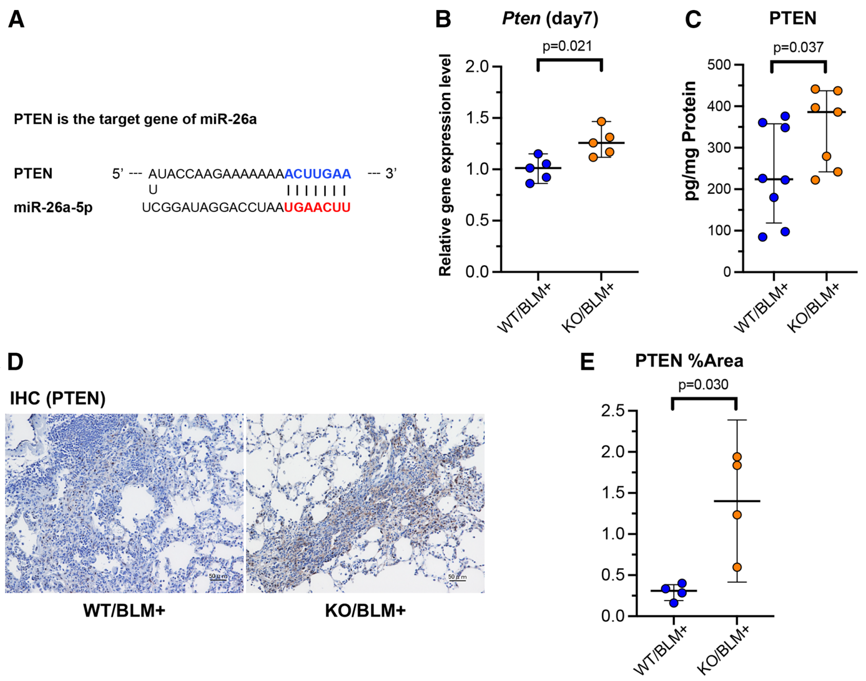

Figure 4. PTEN as a direct target of miR-26a

(A) Sequence alignment showing the complementarity between miR-26a and PTEN. (B) Quantitative PCR analysis of *Pten* mRNA expression in WT and miR-26a KO murine lungs on day 7 after BLM administration (n = 5/group). (C) ELISA analysis of PTEN protein levels in WT and miR-26a KO murine lungs on day 14 after BLM administration (n = 7–8/group). (D) Immunohistochemical staining of WT and miR-26a KO murine lungs on day 14 after BLM administration using anti-PTEN antibodies. Scale bars, 50 μm. (E) Quantification of the stained area (% of total field) in the immunohistochemical staining images shown in Figure 4D (n = 4/group).

our experimental results using identical animal models and fibrosis induction methods as those described in previous reports[5] revealed a significant increase in miR-26a expression levels on day 14 after BLM administration in WT mice (Figure 1C). Furthermore, miR-26a KO mice exhibited significantly lower hydroxyproline levels and milder histological fibrosis after BLM administration (Figures 1D and 1E), contradicting our initial hypothesis. These contrasting results—where pulmonary fibrosis is exacerbated when miR-26a is locally knocked down in the airways but ameliorated when miR-26a is knocked out systemically—suggest that systemic effects may counteract the fibrosis-promoting effects of local miR-26a suppression in the lungs.

As a potential mechanism, we first investigated the role of inflammation, which typically precedes pulmonary fibrosis, to determine whether differences in inflammatory responses between miR-26a KO and WT mice accounted for the observed phenotype. Initially, we confirmed that there were no major abnormalities in the differentiation, maintenance, or distribution of B cells and T cells in KO mice (Figures S1A–G). Furthermore, there was no significant difference in lung structure between the WT and KO groups (Figures S1H and S1I), and whole-lung RNA sequencing also revealed no marked differences (Figure S4). We then considered whether miR-26a KO mice might exhibit reduced susceptibility to fibrosis owing to attenuated systemic inflammation. For instance, elevated serum interleukin (IL)-6, an inflammatory cytokine, has reported to be a predictive marker for acute exacerbation of IPF and a useful indicator of mortality risk.[6] Additionally, IL-6-deficient mice have been shown to exhibit reduced macrophage and neutrophil accumulation following BLM administration, resulting in attenuated pulmonary fibrosis.[7] However, in our study, no significant difference was observed in lung *Il6* mRNA levels between WT and KO mice after BLM administration (Figure 2C). Next, we examined the expression of TGF-β1, a well-known fibrosis-inducing cytokine implicated in various tissues, including the lungs. TGF-β has been shown to induce epithelial-mesenchymal transition in AECs *in vitro*.[8] Moreover, elevated TGF-β expression in the lungs of patients with IPF has been reported, and active TGF-β expression in rat lungs has been demonstrated to induce a pronounced fibrotic response.[9] Based on this, we hypothesized that miR-26a KO mice might exhibit lower TGF-β1 expression in the lungs and serum, resulting in milder fibrosis. However, no significant differences were observed in lung *Tgfb1* mRNA or serum TGF-β1 protein levels between WT and KO mice (Figures 2C and 2D). Additionally, no differences were observed in the proportions of Tregs or CTLs in the lungs after BLM administration (Figure 2B). Taken together, these findings suggest that differences in immune cell populations or inflammation-related factors are unlikely to account for the milder pulmonary fibrosis observed in miR-26a KO mice.

Second, we hypothesized that the absence of suppression of mRNA translation might contribute to the amelioration of fibrosis and conducted a comprehensive investigation using RNA sequencing. PCA of WT and KO mice revealed differences in distribution between the WT and KO groups on day 7 but not differences on day 0 (Figures 3A, 3B, and S4). Hallmark analysis revealed that MTORC1 signaling was elevated in the WT group compared with the KO group (Figure 3C), and KEGG enrichment analysis showed that the PI3K/AKT signaling pathway was enriched in the WT group compared with the KO group (Figure 3D). To further investigate this, we focused on *Timp1*, which is related to fibrosis and is downstream of the PI3K/Akt-mTORC pathway. Four TIMP subtypes (TIMP-1, -2, -3, and -4) are known to inhibit matrix metalloproteinases (MMPs), which are collagen-degrading enzymes.[10,11] TIMPs are produced by AECs and fibroblasts, and their production is enhanced by inflammatory cytokines, including IL-1, tumor necrosis factor alpha (TNF-α), and TGF-β. Although TIMPs are expressed in

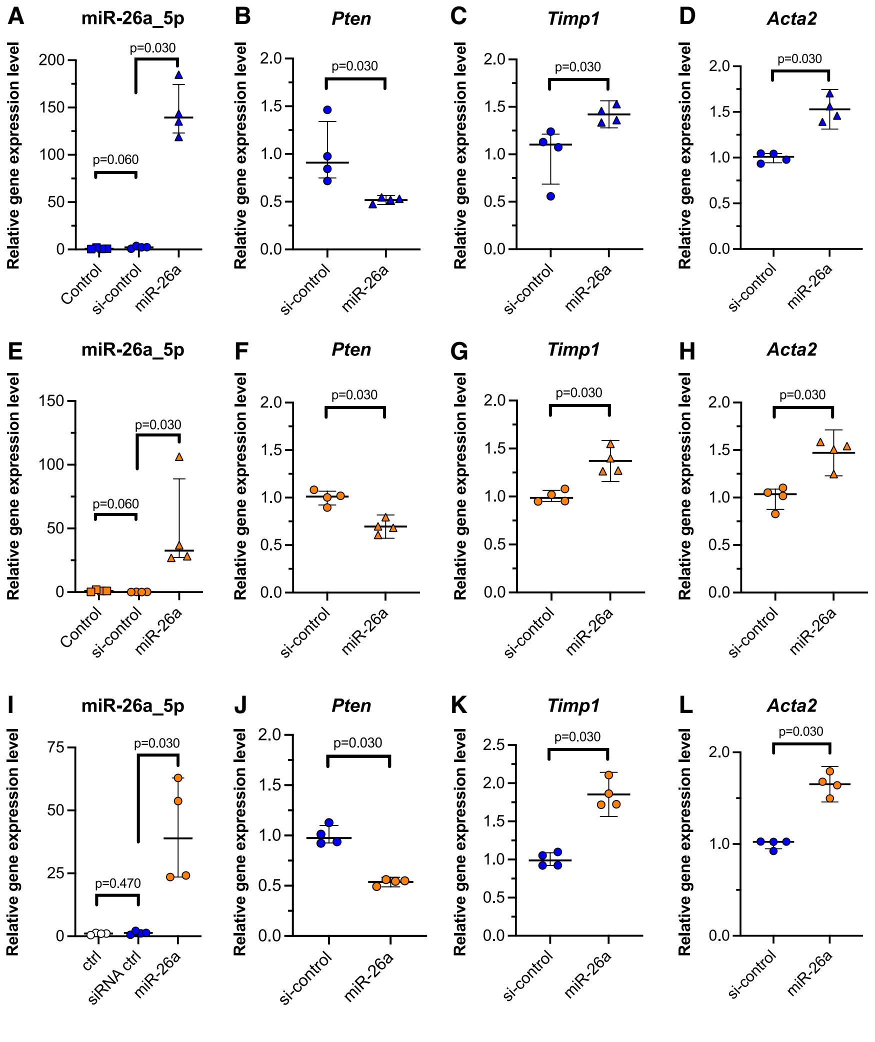

Figure 5. Differences in the effects of miR-26a in mouse-derived cells

(A, E, I) miR-26a levels in primary lung fibroblasts transfected with control, siRNA control, and miR-26a. (A) shows fibroblasts derived from WT mice; (E) shows those from miR-26a KO mice; (I) shows LA-4 cells (n = 4/group). (B–D) Relative changes in *Pten*, *Timp1*, and *Acta2* mRNA expression levels in primary lung fibroblasts derived from WT mice after miR-26a transfection, analyzed using quantitative PCR (n = 4/group). (F–H) Relative changes in *Pten*, *Timp1*, and *Acta2* mRNA expression levels in primary lung fibroblasts derived from miR-26a KO mice after miR-26a transfection, analyzed using quantitative PCR (n = 4/group). (J–L) Relative changes in *Pten*, *Timp1*, and *Acta2* mRNA expression levels in LA-4 cells after miR-26a transfection, analyzed using quantitative PCR (n = 4/group).

many organs throughout the body, TIMP-1 is reported to be particularly abundant in the bladder, lungs, and myocardium (https://www.proteinatlas.org/ENSG00000102265-TIMP1/tissue). Accumulation of extracellular matrix (ECM), a key driver of fibrosis, is regulated by matrix MMPs and their endogenous inhibitors, TIMPs. TIMP-1 is associated with myocardial fibrosis[12] and is used as a biomarker to evaluate the progression of liver fibrosis.[13] Regarding TIMP-1 and pulmonary fibrosis, increased *Timp1* mRNA and protein levels have been reported in the lung tissue and BALF following intratracheal BLM administration in C57BL/6 mice.[14] A study comparing fibrosis-sensitive C57BL/6 mice and fibrosis-resistant BALB/c mice showed that *Timp1* mRNA levels and BALF concentrations were significantly elevated 1 and 14 days after BLM administration compared with pre-administration levels, with significantly higher levels in the C57BL/6 group. Thus, elevated TIMP-1 levels are strongly correlated with the severity of fibrosis.[15] Additionally, increased *Timp1* mRNA levels were observed in lung tissues obtained from patients with IPF in the IPF-PRO Registry cohort, where elevated levels were associated with disease severity, as measured using pulmonary function tests and imaging-based fibrosis assessment.[16] While TIMP-1 appears to play a significant role in the mechanism by which miR-26a KO mice exhibited milder pulmonary fibrosis than WT mice (Figures 3E–3I), *Timp1* does not contain a direct target sequence for miR-26a. Therefore, we explored the potential upstream pathway target genes. In neonatal cardiac fibroblasts, inhibition of the PI3K/Akt-mTOR signaling pathway suppresses the transcription and translation of *Timp1*, *Col1*, and *Tgfb1*, suggesting that the mTOR pathway regulates *Timp1* expression.[17] Keith et al. have reported that mTOR inhibitors inhibit human lung myofibroblast differentiation, resulting in ECM deposition.[18] Through an analysis of miR-26a target genes related to the mTOR pathway, we identified the *Pten* as a potential upstream regulator. miR-26a is known to suppress *Pten* translation by targeting its 3′ UTR[19] (TargetScan, https://www.targetscan.org/vert_80/) (Figure 4A). PTEN inhibits the PI3K-Akt-mTOR pathway, which regulates cell proliferation, survival, and energy homeostasis.[20] The suppression of *Pten* by miR-26a inhibits the mTOR pathway, thereby reducing *Timp1* expression.[17] AEC injury and subsequent repair abnormalities are crucial in the progression of pulmonary fibrosis, with injured AECs releasing pro-fibrotic signals, including TGF-β, that activate fibroblasts and promote ECM accumulation.[21] Miyoshi et al. have reported that lung-epithelium-specific PTEN-deficient mice exhibited exacerbated fibrosis after BLM administration compared with WT mice.[22] These mice exhibited increased AEC dissociation, decreased tight junction protein expression, and enhanced basement membrane disruption after lung injury, suggesting that epithelial PTEN plays a protective role in maintaining the integrity of the alveolar epithelium. Moreover, decreased *Pten* expression and Akt activation were observed in the AECs obtained from patients with IPF, further

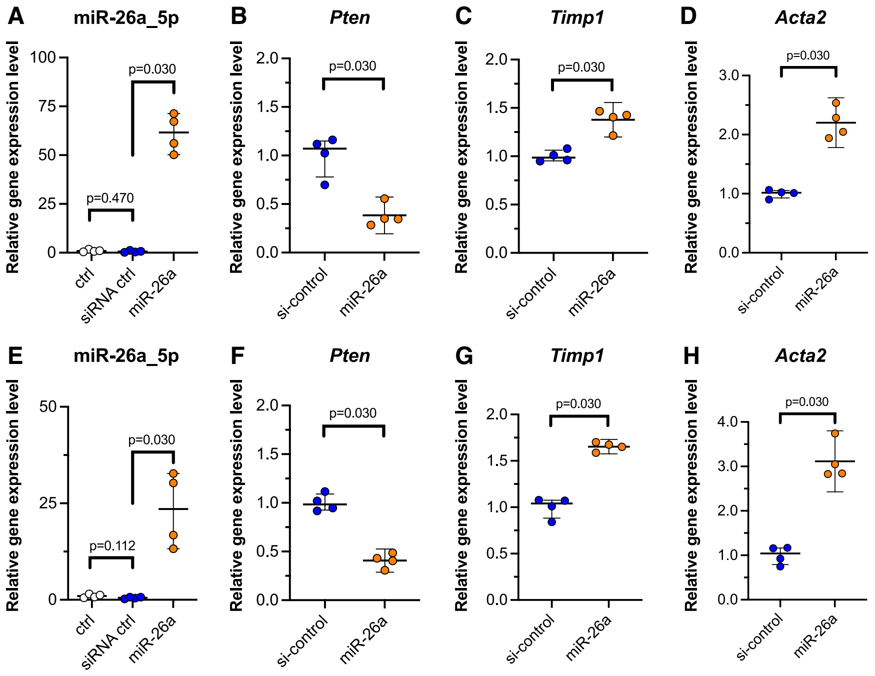

Figure 6. Differences in the effects of miR-26a in human-derived cells

(A and E) miR-26a levels in MRC-5 cells and A549 cells transfected with control, siRNA control, and miR-26a. (A) shows MRC-5 cells; (E) shows A549 cells (n = 4/group).

(B–D) Relative changes in *Pten*, *Timp1*, and *Acta2* mRNA expression levels in MRC-5 cells after miR-26a transfection, analyzed using quantitative PCR (n = 4/group).

(F–H) Relative changes in *Pten*, *Timp1*, and *Acta2* mRNA expression levels in A549 cells after miR-26a transfection, analyzed using quantitative PCR (n = 4/group).

indicating the importance of PTEN in the pathogenesis of pulmonary fibrosis. In our study, *Pten* mRNA and protein levels were significantly increased in the KO group compared with those in the WT group following BLM administration (Figures 4B–4E). Furthermore, transfection of miR-26a into fibroblasts derived from both WT and KO mice decreased *Pten* expression and increased *Timp1* and *Acta2* expression (Figure 5). Additionally, miR-26a KO mice showed milder epithelial cell damage or faster recovery compared with WT mice (Figure S6), reflecting the protective effects of PTEN on AECs. Based on these results, the mechanism by which miR-26a KO mice exhibited milder pulmonary fibrosis than WT mice is summarized in graphical abstract. Previous reports have suggested that PTEN inhibits the PI3K-Akt-mTOR pathway,[17,20] which exerts protective effects on AECs,[22,23] and that TIMP-1 promotes ECM accumulation, including that of type I collagen.[24] Under normal conditions, the PI3/Akt-mTOR pathway is activated by cytokines such as TGF-β1, leading to increased TIMP-1 expression and promoting fibrosis through ECM accumulation. However, in miR-26a KO mice, elevated Pten expression suppresses the mTOR pathway, leading to reduced Timp1 expression and myofibroblast differentiation, without changes in TGF-β protein or mRNA levels, ultimately resulting in decreased ECM accumulation and attenuated fibrosis. These findings suggest that the PTEN/TIMP-1 axis acts as a TGF-β-independent pathway contributing to fibrosis regulation in miR-26a KO mice. Additionally, enhanced *Pten* expression in miR-26a KO mice may reduce AEC damage, contributing to the attenuation of subsequent pulmonary fibrosis.

The results of our study differed from those of a previous report,[5] which concluded that miR-26a ameliorates pulmonary fibrosis. However, consistent with the findings of the present study, more recent research using miR-26a KO mice has reported attenuated fibrosis in the shoulder joint in a frozen shoulder model compared with WT mice, along with reduced expression levels of *Col1a1* in synovial fibroblasts.[25] There are several possible explanations for the discrepancy. First, approximately 1,000 target genes have been reported for miR-26a (TargetScan, http://www.targetscan.org/), and it is possible that multiple genes other than *Pten* are affected. Additionally, as multiple miRNAs share target mRNAs with complementary sequences, the expression levels of individual miRNAs may directly influence each other or shared signaling pathways.[26] For instance, both miR-21 and miR-146a regulate the nuclear factor-κB signaling pathway, and their balance affects the inflammatory response.[27] Furthermore, miRNA functions are modulated through complex interactions with other miRNAs and their mRNA targets, and altered miRNA expression levels have also been shown to affect target selection.[28]

In the case of miR-26a KO, as in this study, the relative influence of other miRNAs may have increased or decreased, potentially contributing to the observed improvement in fibrosis. However, our findings revealed that the expression levels of miR-21, miR-29, and miR-199a, which have been implicated in the regulation of pulmonary fibrosis, were significantly elevated 14 days after BLM administration in WT mice (Figure S1J), whereas no significant changes were detected in miR-26a KO mice (Figure S1K). These results suggest that the attenuation of fibrosis observed in miR-26a KO mice is primarily attributable to the deficiency of miR-26a itself rather than secondary changes in other miRNAs. Conversely, these findings suggest that BLM administration induces changes in the expression of multiple miRNAs, which may interact with common fibrotic signaling pathways. Second, miR-26a exerts cell-type-specific effects. For example, miR-21a has been shown to increase collagen cross-linking in cardiac fibroblasts and cause myocardial fibrosis, while exhibiting cardioprotective effects by inhibiting cardiomyocyte apoptosis.[29] Compared with the localized suppression or overexpression of miR-26a in the airways and lungs, systemic KO of miR-26a may

have resulted in more dynamic changes. According to the single-cell transcriptomic data of human lung cells from patients with IPF reported by Adams et al.,[30] *Pten* is broadly expressed in both epithelial cells and fibroblasts, whereas *Timp1* is mainly expressed in fibroblasts. These findings may explain the differences between the previously reported miR-26a agonist/antagonist models, which primarily act on airway epithelial cells, and the global KO model that affects both epithelial cells and fibroblasts (Figure S7).

Finally, differences in the timing of miR-26a suppression may have influenced our results. In BLM-induced pulmonary fibrosis, it has been reported that early suppression of IL-6 worsens fibrosis, while late suppression improves it.[31] This suggests that the timing of the miR-26a suppression plays a critical role. In miR-26a KO mice, suppression occurred before the onset of pulmonary fibrosis, whereas the suppression of miR-26a using antagomiR occurred after BLM administration. These differences in timing may have contrasting effects on the progression of fibrosis. Further investigation involving the temporal modulation of miR-26a, either through overexpression or inhibition, at multiple time points following BLM administration, may provide a more detailed understanding of its role during the progression of fibrosis. Potential therapeutic approaches based on this mechanism include the use of miR-26a antagomiRs, small molecules that stabilize or activate PTEN, and the targeted delivery of RNA-based therapeutics. Although preclinical researches have advanced our understanding of these mechanisms, the field of miRNA-based therapeutics remains in its early stages, with only a few studies progressing to clinical development.[32]

The limitations of this study were as follows. Although miR-26a is a common miRNA present in both mice and humans and we also obtained supportive results in human cell lines, our main findings were derived from mouse experiments, and their relevance to human disease remains to be clarified. Additionally, the *in vivo* effects of miR-26a on different organs and cell types have not yet been fully elucidated. Moreover, because the KO mice were bred in our facility, the number of mice available at one time was limited, leading to experiments with relatively small sample sizes. Finally, miRNA-mediated regulatory networks are highly complex. The PTEN and PI3K/Akt-mTOR signaling pathways examined in this study represent only a part of this intricate network, and other relevant pathways may play additional roles.

Conclusions

We demonstrated that miR-26 KO enhances PTEN expression and decreases downstream TIMP-1 expression, thereby attenuating pulmonary fibrosis in miR-26a KO mice. Although numerous studies have shown that miR-26a acts in a lung-protective manner, this study is the first to present findings suggesting the opposite effect.

The contrasting outcomes between local and systemic suppression of miR-26a, as observed in our study, suggest that systemic suppression of miR-26a across multiple organs and cell types may result in entirely different mechanisms than localized suppression. Elucidating the specific roles of individual miRNAs is essential for identifying potential therapeutic targets; however, to translate these findings into clinical applications, further studies using conditional knockout models targeting specific cell types or organs, miRNA profiling, and network analyses will be required.

These findings further highlight the need to overcome key challenges, such as improving miRNA selectivity toward their intended targets and optimizing the timing of intervention before miRNA-based therapies can be translated into clinical applications. Elucidating the intricate regulatory networks mediated by miRNAs is critical for understanding disease mechanisms and developing novel therapies.

MATERIALS AND METHODS

Animals

miR-26a KO, C57BL/6J, and BALB/cJ mice were used in this experiment. In comparative experiments with miR-26a KO mice, littermates without mutations were used as the control group and referred to as WT. miR-26a-1 KO and miR-26a-2 KO mice were generated individually using the CRISPR/Cas9 system, as previously described.[33,34] Briefly, two single-guide RNAs targeting each of the miR-26a-1 and miR-26a-2 genes were designed to form a complex with the genome-editing enzyme Cas9. This complex recognizes and cleaves target genes, resulting in a KO state. Heterozygous mice with the same deletion sequence were bred to generate homozygous KO mice (miR-26a-1$^{-/-}$ and miR-26a-2$^{-/-}$). Subsequently, these homozygous KO mice were crossed to produce complete miR-26a KO mice (miR-26a$^{-/-}$). To confirm the gene deletion, genotyping PCR was performed using tail DNA and the following primer sets: miR-26a-1: F, CTGCCTGTTGTTGTCTA; R, CTACAG GCAAAGGTTGAGA; and miR-26a-2: F, CatAGACTGGTGGCC AGTT; R, CTTCATTGAGGCAGACCAT. The absence of miR-26a expression in the tissues and organs of the miR-26a KO mice was confirmed using PCR. C57BL/6J and BALB/cJ mice (6–8 weeks old, male) were purchased from The Jackson Laboratory Japan (Yokohama, Japan) and housed in a pathogen-free room under controlled environmental conditions with a 12-h light/dark cycle and free access to food and water. All experimental procedures were approved by the Animal Research Committee of Hiroshima University (approval numbers: A22-86 and 2021-51-7) and were conducted in accordance with the *Guide for the Care and Use of Laboratory Animals*, 8th ed., 2010 (National Institutes of Health, Bethesda, MD, USA).

OA of BLM in mice

Eight-week-old male mice were randomly divided into the BLM-induced pulmonary fibrosis group and control groups. To induce the pulmonary fibrosis, each mouse was anesthetized via intraperitoneal injection of 100 μL of a mixed anesthetic containing medetomidine (0.3 mg/kg body weight, Kyoritsu Seiyaku, Tokyo, Japan), midazolam (4 mg/kg body weight, Sandoz K.K., Tokyo, Japan), and butorphanol (5 mg/kg body weight, Meiji Seika Pharma, Tokyo, Japan). BLM (Nippon Kayaku, Tokyo, Japan) diluted with saline

was administered oropharyngeally using a micropipette, with a dosage of 1.5 μg/g body weight. The mice in the control group were administered saline. Following BLM administration, mice were revived from anesthesia with an intraperitoneal injection of 100 μL of atipamezole hydrochloride (Kyoritsu Seiyaku). On days 7 and 14 after BLM administration, mice were re-anesthetized using the same anesthetic protocol. BALF and lungs were collected for further analysis. These analyses included measurement of hydroxyproline content, mRNA expression analysis, flow cytometry, ELISA, histological evaluation, and whole-lung RNA sequencing.

Hydroxyproline assay

The left lung was used for the hydroxyproline analysis. The hydroxyproline measurement was performed as follows: The left lung was homogenized in 1 mL of phosphate-buffered saline (PBS), and 1 mL of HCl was added for hydrolysis at 120°C for 16 h. After hydrolysis, 500 μL of the supernatant was centrifuged at 10,000 rpm for five minutes. From this, 5 μL of the supernatant was transferred into a 96-well plate. Hydroxyproline standards (5 μL) were also added to the 96-well plate, followed by the addition of 5 μL of citrate/acetate buffer and 100 μL of chloramine T solution. After incubation at room temperature for 30 min, 100 μL of Ehrlich's reagent was added, and the plate was further incubated at 65°C for 30 min. After cooling to room temperature for 5 min, the absorbance was measured at 550 nm using a plate reader.

Analysis of BALF

BALF was collected on day 0 (before BLM OA) and on days 3, 7, and 14 after BLM OA. After euthanasia, the trachea of each mouse was exposed and cannulated using an 18-gauge cannula. The lungs were washed three times with PBS (0.5 mL), and the lavage fluid was collected. After centrifuging at 300 × *g* for five minutes at 4°C to remove cells, the supernatant was stored at −80°C. The cell pellet was resuspended in 1 mL of Dulbecco's modified Eagle's medium (DMEM), and red blood cells were lysed using ACK lysis buffer (Thermo Fisher Scientific, Waltham, MA, USA). Total cell counts were determined using an automated cell counter, whereas differential cell counts were assessed using Diff-Quik staining (International Reagents, Kobe, Japan) after cytospin preparation (Thermo Fisher Scientific).

Blood sampling

Blood samples for TGF-β1 measurement were collected from the facial vein or via cardiac puncture of the right ventricle of anesthetized mice on days 0 (before BLM OA), 7, and 14 after BLM OA. The collected blood samples were centrifuged at 3,000 rpm for 15 min at 4°C to remove cells, and the serum was stored at −80°C.

Histological examination

The collected lung tissue sections were fixed in 2% formalin solution (Nacalai Tesque, Kyoto, Japan), embedded in paraffin, and stained with H&E and Azan stain for histological analysis. The equivalent diameter of the alveolar wall was measured as described previously.[35] Four randomly selected regions were imaged and captured at a magnification of ×40 using a BZ-9000 microscope (Keyence, Osaka, Japan), and a 10-μm division calibration slide (AX0001, Olympus Co., Tokyo, Japan) was used to generate scale bars. Images were quantified using ImageJ2 (v.2.16.0/1.54p).[36] All images were calibrated using the same scale. Eight measurements of alveolar wall thickness were recorded for each image.

Quantitative PCR

The extracted murine lungs were homogenized in 1 mL of TRIzol reagent (Life Technologies), and RNA was extracted using the RNeasy Mini Kit (QIAGEN, Venlo, the Netherlands). The extracted RNA was reverse transcribed into cDNA using a High-Capacity RNA-to-cDNA Kit (Applied Biosystems, Foster City, CA, USA). The expressions of mRNA were measured using quantitative PCR with the Applied Biosystems 7500 Fast Real-Time PCR System (Applied Biosystems) with the following TaqMan Gene Expression Assays (all from Applied Biosystems) according to the manufacturer's protocol: *Tgfb1* (Assay ID, Mm03024053_m1), *Il6* (Assay ID, Mm00446190_m1), *Pten* (Assay ID, Mm00477208_m1), *Timp1* (Assay ID, Mm01341361_m1), *Col1a1* (Assay ID, Mm00801666_g1), *Acta2* (Assay ID, Mm00725412_s1), *Pten* (Assay ID, Hs02621230_s1), *Timp1* (Assay ID, Hs01092512_g1), and *Acta2* (Assay ID, Hs00426835_g1). The expression of miR-26a-5p (Assay ID, RT/TM000405), miR-21 (Assay ID, 000397), miR-29a (Assay ID, 002112), and miR-199a-3p (Assay ID, 002304) was measured using quantitative PCR with a TaqMan miRNA assay (Thermo Fisher Scientific). *Rn18s* (Assay ID, Mm03928990_g1), *Actb* (Assay ID, Mm02619580_g1), *Rn18s* (Assay ID, Hs99999901_s1), and *Actb* (Assay ID, Hs01060665_g1) for the gene and U6 snRNA (Assay ID, RT/TM001973) for the miRNA were used as internal controls to normalize the sample differences.

ELISA

Serum levels of TGF-β1 were measured by using ELISA with the TGF-β1 ELISA Kit (R&D Systems) according to the manufacturer's protocol. The total protein concentration in the samples was also measured, and the results were expressed as ng/mg of protein. Using tissue inhibitor of metalloproteinase-1 (TIMP-1)-Cell Lysate Mouse ELISA kit (Thermo Fisher Scientific) and the ELISA Kit for Phosphatase and Tensin Homolog (PTEN) WLS/96test (Cosmo Bio), TIMP-1 and PTEN protein levels were measured in homogenized right lung lobes of the mice. The results were expressed as ng/mg of protein for TIMP-1 and pg/mg of protein for PTEN. TIMP-1 protein levels in the BALF were measured and expressed as ng/mL.

RNA extraction and sequencing

WT and KO mice were euthanized on day 0 (before BLM OA) and 7 days after BLM OA. The extracted murine lungs were homogenized in 1 mL of TRIzol reagent (Life Technologies), and total lung RNA was extracted using the RNeasy Mini Kit according to the manufacturer's protocol. Transcriptome analysis, based on 3′ UTR RNA sequencing, was performed at the Department of Trans-omics, Medical Institute of Bioregulation, Kyushu University (Fukuoka, Japan).[37] Transcriptome data were analyzed using the

integrated differential expression and pathway (iDEP) platform[38] to identify differentially expressed genes and to perform pathway enrichment analysis based on the KEGG and HALLMARK gene sets. Raw RNA sequencing data were deposited in the NCBI database for the Biotechnology Information Gene Expression Omnibus database (GEO: GSE302309).

Immunostaining

Lung tissue sections were subjected to antigen retrieval in 10 mmol/L sodium citrate buffer (pH 6.0) in a microwave for 20 min, followed by staining with the ENVISION+kit/horseradish peroxidase (HRP) (Dako, Tokyo, Japan).[39] After blocking endogenous peroxidase and proteins, the sections were incubated with rabbit anti-TIMP-1 antibody (BS-0415R, 1:200, Bioss, Woburn, MA, USA) and rabbit anti-PTEN antibody (#9559, 1:1,000; CST, USA). Next, HRP-labeled anti-rabbit immunoglobulin G (IgG) antibody (#424144, Nichirei, Tokyo, Japan) was used, followed by a substrate chromogen reaction. Sections were counterstained with hematoxylin. The stained specimens were checked, and micrographs were obtained under the same photographic conditions at four representative locations where DAB coloration was considered the strongest. The micrographs were captured using a BZ-9000 microscope. The ImageJ2 was used to separate the DAB- and hematoxylin-stained regions. The percentage areas of the DAB-stained regions were calculated and compared.

Cell isolation

LA-4 cells, A549 cells, and MRC5 cells were cultured in DMEM (Thermo Fisher Scientific) supplemented with 10% fetal bovine serum and 1% penicillin/streptomycin and maintained at 37°C in a 5% CO_2 incubator. To isolate primary lung fibroblasts, WT and KO mice were euthanized and perfused with 10 mL of PBS via the right ventricle. The whole lung was excised, finely minced, and incubated with shaking at 37°C for 30 min in RPMI 1640 medium (Thermo Fisher Scientific) containing 1.0 mg/mL of collagenase A (Roche Diagnostics, Basel, Switzerland) to generate a single-cell suspension. The resulting solution was filtered through 100 and 40 μm filters to remove debris, and the cell suspension was centrifuged at 1,200 rpm for four minutes at 4°C to obtain a cell pellet. The cell pellet was resuspended in DMEM supplemented with 10% fetal bovine serum and 1% penicillin/streptomycin. After counting, the cells were seeded at a density of 2.5×10^6 cells per 10 cm dish and cultured at 37°C in a 5% CO_2 incubator. The first medium change was performed 24 h after seeding, and the medium was subsequently changed every 2–3 days for a total of three passages.

Flow cytometry

Single-cell suspensions for flow cytometry were prepared as follows. The lower right lobe of the mouse lung was excised and finely minced. The lung tissue was incubated with shaking at 37°C for 30 min in RPMI 1640 medium containing 1.0 mg/mL collagenase A. The resulting suspension was passed through a 70 μm filter to remove debris, and red blood cells were removed using ACK lysis buffer. The cell suspension was blocked with anti-CD16/32 antibody (FcγR, clone 93, BioLegend, San Diego, CA, USA) to prevent non-specific binding and then incubated with fluorescently labeled antibodies diluted to the appropriate concentrations. The following rat monoclonal antibodies were used: CD31-PerCP (clone 390), CD45-APC (clone 30-F11), EpCAM-PB (clone G8.8), CD3 (clone 17A2), CD4 (clone GK1.5), CD8 (clone 53–6.7), and CD25 (clone PC61) (all from BioLegend). Cell analysis was performed using a BD FACS Aria II (BD Biosciences, Franklin Lakes, NJ, USA) or BD LSR Fortessa X-20 (BD Biosciences), and the data were analyzed using FlowJo software (Tree Star, Ashland, OR, USA). For immune cell analysis, single-cell suspensions from the spleen, bone marrow, and thymus were resuspended in Gey's solutions to lyse red blood cells. Cells were treated with TruStain FcX (93, BioLegend) followed by staining with fluorochrome-conjugated antibodies or biotinylated monoclonal antibodies CD19 (clone 6D5), B220 (clone RA3-6B2), IgD (clone 11-26c.2a), IgM (clone RMM-1), CD5 (clone 53–7.3), CD21 (clone 7E9), CD38 (clone 90), CD73 (clone TY/11.8), CD138 (clone 281–2), and TACI (clone 8F10) from BioLegend; CD23 (clone B3B4) from Invitrogen; CD25 (clone REAL537) from Miltenyi Biotec; and AA4.1 (clone 493), Fas (clone SA367H8), CD4 (clone RM4-5), CD8 (clone 53–6.7), CD44 (clone IM7), and CD62L (clone MEL-14) from BD Biosciences. APC-conjugated streptavidin (BD Biosciences) and BV650-conjugated streptavidin (BioLegend) were used to detect the biotin-marked cells. FACS analysis was performed using CytoFLEX S (Beckman Coulter, Brea, CA, USA), and the data were analyzed using FlowJo software.

miR-26a transfection

miR-26a was transfected into primary lung fibroblasts and the mouse epithelial cell line LA-4, the human alveolar epithelial cell line A549, and the human lung fibroblast cell line MRC-5 to measure the mRNA expression levels of *Pten*, *Timp1*, *Col1a1*, and *Acta2* using quantitative PCR. Primary lung fibroblasts and LA-4 cells were seeded in 24-well plates at a density of 3×10^4 cells per well. For transfection, 25 μL of mmu-miR-26a, a synthetic miRNA (Hokkaido System Science, Sapporo, Japan) or small interfering RNA (siRNA) was mixed with Opti-MEM Reduced Serum Medium (Thermo Fisher Scientific, ID: 31985062). Separately, 25 μL of Lipofectamine RNAiMAX Transfection Reagent (Thermo Fisher Scientific, ID: 13778150) was also mixed with Opti-MEM Reduced Serum Medium. These two solutions were combined to create a final volume of 50 μL per well. Next, 450 μL of DMEM was added to each well along with 50 μL of the reagent solutions. The plate was gently shaken to ensure proper mixing, and the cells were incubated at 37°C. After 24 h, the medium was replaced with fresh DMEM. After an additional 24-h incubation, the wells were washed with PBS. TRIzol was added to collect the cells and extract RNA.

Statistical analysis

Statistical analyses were performed using JMP Pro 16 software (SAS Institute, Cary, NC, USA). The Wilcoxon/Kruskal-Wallis test was used to evaluate the statistical significance between groups. p values < 0.05 were considered statistically significant.

DATA AND CODE AVAILABILITY

The datasets used and analyzed in the present study are available from the corresponding author upon reasonable request.

ACKNOWLEDGMENTS

The authors declare that this research was self-funded and did not receive financial support from any funding agency, institutions, or commercial entity. We would like to thank Ms. T. Miyata and E. Ueda (Department of Orthopedic Surgery, Graduate School of Biomedical and Health Sciences, Hiroshima University) for breeding and providing miR-26a KO mice and for excellent technical support and Ms. Y Hayashi (Natural Science Center for Basic Research and Development, Hiroshima University) for technical assistance with flow cytometry analysis. We would also like to thank Editage (www.editage.com) for English language editing. During the preparation of this work the authors used ChatGPT (GPT-4o, OpenAI) to enhance readability and proofread the English text. After using this service, the authors reviewed and edited the content as needed and take full responsibility for the content of the publication. Part of this study was performed at the Research Facilities for Laboratory Animal Science. This work was partially supported by the Natural Science Center for Basic Research and Development (NBARD-00093), Program of the Network-type Joint Usage/Research Center for Radiation Disaster Medical Science, and JSPS Program for Forming Japan's Peak Research Universities (J-PEAKS) grant number JPJS00420230011.

AUTHOR CONTRIBUTIONS

A.H., K.S., and T.N. conceptualized the study; A.H., K.S., and T.N. designed the study; A.H., K.S., T.N., Y.G., T.Y., and S.M. performed the experiments; A.H., K.S., T.N., Y.G., and T.Y. prepared the figures; T.N. and N.H. acquired funding; A.H., K.S., and T.N. managed the study; N.H. supervised the study; A.H., K.S., T.N., and S.M. drafted the manuscript; and K.Y., S.S., Y.H., T.M., H.I., H.H., Y.G., T.Y., and N.H. reviewed and edited the manuscript. All the authors have read and approved the final version of the manuscript.

DECLARATION OF INTERESTS

The authors declare no competing interests.

SUPPLEMENTAL INFORMATION

Supplemental information can be found online at https://doi.org/10.1016/j.omtn.2025.102765.

REFERENCES

1. Li, H., Zhao, X., Shan, H., and Liang, H. (2016). MicroRNAs in idiopathic pulmonary fibrosis: involvement in pathogenesis and potential use in diagnosis and therapeutics. Acta Pharm. Sin. B *6*, 531–539. https://doi.org/10.1016/j.apsb.2016.06.010.

2. Yang, T., Wang, J., Zhao, J., and Liu, Y. (2022). Current and prospective applications of exosomal microRNAs in pulmonary fibrosis (Review). Int. J. Mol. Med. *49*, 37. https://doi.org/10.3892/ijmm.2022.5092.

3. Cadena-Suárez, A.R., Hernández-Hernández, H.A., Alvarado-Vásquez, N., Rangel-Escareño, C., Sommer, B., and Negrete-García, M.C. (2022). Role of MicroRNAs in Signaling Pathways Associated with the Pathogenesis of Idiopathic Pulmonary Fibrosis: A Focus on Epithelial-Mesenchymal Transition. IJMS *23*, 6613. https://doi.org/10.3390/ijms23126613.

4. Yano, H., Hamanaka, R., Zhang, J.J., Yano, M., Hida, M., Matsuo, N., and Yoshioka, H. (2021). MicroRNA-26 regulates the expression of CTGF after exposure to ionizing radiation. Radiat. Environ. Biophys. *60*, 411–419. https://doi.org/10.1007/s00411-021-00915-9.

5. Liang, H., Xu, C., Pan, Z., Zhang, Y., Xu, Z., Chen, Y., Li, T., Li, X., Liu, Y., Huangfu, L., et al. (2014). The Antifibrotic Effects and Mechanisms of MicroRNA-26a Action in Idiopathic Pulmonary Fibrosis. Mol. Ther. *22*, 1122–1133. https://doi.org/10.1038/mt.2014.42.

6. Lee, J.H., Jang, J.H., Park, J.H., Jang, H.-J., Park, C.S., Lee, S., Kim, S.-H., Kim, J.Y., and Kim, H.K. (2021). The role of interleukin-6 as a prognostic biomarker for predicting acute exacerbation in interstitial lung diseases. PLoS One *16*, e0255365. https://doi.org/10.1371/journal.pone.0255365.

7. Saito, F., Tasaka, S., Inoue, K.I., Miyamoto, K., Nakano, Y., Ogawa, Y., Yamada, W., Shiraishi, Y., Hasegawa, N., Fujishima, S., et al. (2008). Role of Interleukin-6 in Bleomycin-Induced Lung Inflammatory Changes in Mice. Am. J. Respir. Cell Mol. Biol. *38*, 566–571. https://doi.org/10.1165/rcmb.2007-0299OC.

8. Willis, B.C., Liebler, J.M., Luby-Phelps, K., Nicholson, A.G., Crandall, E.D., and Borok, Z. (2005). Induction of epithelial-mesenchymal transition in alveolar epithelial cells by transforming growth factor-β1. Am. J. Pathol. *166*, 1321–1332. https://doi.org/10.1016/s0002-9440(10)62351-6.

9. Zhao, J., Shi, W., Wang, Y.-L., Chen, H., Bringas, P., Datto, M.B., Frederick, J.P., Wang, X.-F., and Warburton, D. (2002). Smad3 deficiency attenuates bleomycin-induced pulmonary fibrosis in mice. Am. J. Physiol. Lung Cell. Mol. Physiol. *282*, L585–L593. https://doi.org/10.1152/ajplung.00151.2001.

10. Vandenbroucke, R.E., and Libert, C. (2014). Is their new hope for therapeutic matrix metalloproteinase inhibition? Nat. Rev. Drug Discov. *13*, 904–927. https://doi.org/10.1038/nrd4390.

11. Ries, C. (2014). Cytokine functions of TIMP-1. Cell. Mol. Life Sci. *71*, 659–672. https://doi.org/10.1007/s00018-013-1457-3.

12. López, B., González, A., and Díez, J. (2010). Circulating Biomarkers of Collagen Metabolism in Cardiac Diseases. Circulation *121*, 1645–1654. https://doi.org/10.1161/CIRCULATIONAHA.109.912774.

13. Abignano, G., Blagojevic, J., Bissell, L.-A., Dumitru, R.B., Eng, S., Allanore, Y., Avouac, J., Bosello, S., Denton, C.P., Distler, O., et al. (2019). European multicentre study validates enhanced liver fibrosis test as biomarker of fibrosis in systemic sclerosis. Rheumatology *58*, 254–259. https://doi.org/10.1093/rheumatology/key271.

14. Madtes, D.K., Elston, A.L., Kaback, L.A., and Clark, J.G. (2001). Selective Induction of Tissue Inhibitor of Metalloproteinase-1 in Bleomycin-Induced Pulmonary Fibrosis. Am. J. Respir. Cell Mol. Biol. *24*, 599–607. https://doi.org/10.1165/ajrcmb.24.5.4192.

15. Manoury, B., Caulet-Maugendre, S., Guénon, I., Lagente, V., and Boichot, E. (2006). Timp-1 is a Key Factor of Fibrogenic Response to Bleomycin in Mouse Lung. Int. J. Immunopathol. Pharmacol. *19*, 471–487. https://doi.org/10.1177/039463200601900303.

16. Todd, J.L., Vinisko, R., Liu, Y., Neely, M.L., Overton, R., Flaherty, K.R., Noth, I., Newby, L.K., Lasky, J.A., Olman, M.A., et al. (2020). Circulating matrix metalloproteinases and tissue metalloproteinase inhibitors in patients with idiopathic pulmonary fibrosis in the multicenter IPF-PRO Registry cohort. BMC Pulm. Med. *20*, 64. https://doi.org/10.1186/s12890-020-1103-4.

17. Magaye, R.R., Savira, F., Hua, Y., Xiong, X., Huang, L., Reid, C., Flynn, B.L., Kaye, D., Liew, D., and Wang, B.H. (2021). Attenuating PI3K/Akt- mTOR pathway reduces dihydrosphingosine 1 phosphate mediated collagen synthesis and hypertrophy in primary cardiac cells. Int. J. Biochem. Cell Biol. *134*, 105952. https://doi.org/10.1016/j.biocel.2021.105952.

18. Ferguson, K.T., Torr, E.E., Bernau, K., Leet, J., Sherris, D., and Sandbo, N. (2017). The Novel mTOR Complex 1/2 Inhibitor P529 Inhibits Human Lung Myofibroblast Differentiation. J. Cell. Biochem. *118*, 2241–2249. https://doi.org/10.1002/jcb.25878.

19. Li, B., and Sun, H. (2013). miR-26a promotes neurite outgrowth by repressing PTEN expression. Mol. Med. Rep. *8*, 676–680. https://doi.org/10.3892/mmr.2013.1534.

20. Ghafouri-Fard, S., Abak, A., Shoorei, H., Mohaqiq, M., Majidpoor, J., Sayad, A., and Taheri, M. (2021). Regulatory role of microRNAs on PTEN signaling. Biomed. Pharmacother. *133*, 110986. https://doi.org/10.1016/j.biopha.2020.110986.

21. Zhu, W., Tan, C., and Zhang, J. (2022). Alveolar Epithelial Type 2 Cell Dysfunction in Idiopathic Pulmonary Fibrosis. Lung *200*, 539–547. https://doi.org/10.1007/s00408-022-00571-w.

22. Miyoshi, K., Yanagi, S., Kawahara, K., Nishio, M., Tsubouchi, H., Imazu, Y., Koshida, R., Matsumoto, N., Taguchi, A., Yamashita, S.i., et al. (2013). Epithelial Pten Controls Acute Lung Injury and Fibrosis by Regulating Alveolar Epithelial Cell Integrity. Am. J. Respir. Crit. Care Med. *187*, 262–275. https://doi.org/10.1164/rccm.201205-0851OC.

23. Qiu, T., Tian, Y., Gao, Y., Ma, M., Li, H., Liu, X., Wu, H., Zhang, Y., Ding, H., Cao, M., et al. (2019). PTEN loss regulates alveolar epithelial cell senescence in pulmonary fibrosis depending on Akt activation. Aging *11*, 7492–7509. https://doi.org/10.18632/aging.102262.

24. Verrecchia, F., and Mauviel, A. (2007). Transforming growth factor-β and fibrosis. WJG *13*, 3056–3062. https://doi.org/10.3748/wjg.v13.i22.3056.

25. Sumimoto, Y., Harada, Y., Yimiti, D., Watanabe, C., Miyaki, S., and Adachi, N. (2024). MicroRNA-26a deficiency attenuates the severity of frozen shoulder in a mouse immobilization model. J. Orthop. Res. *42*, 2623–2633. https://doi.org/10.1002/jor.25940.

26. Dai, B., Wang, F., Nie, X., Du, H., Zhao, Y., Yin, Z., Li, H., Fan, J., Wen, Z., Wang, D.W., and Chen, C. (2020). The Cell Type–Specific Functions of miR-21 in Cardiovascular Diseases. Front. Genet. *11*, 563166. https://doi.org/10.3389/fgene.2020.563166.

27. Hill, M., and Tran, N. (2021). miRNA interplay: mechanisms and consequences in cancer. Dis. Model. Mech. *14*, dmm047662. https://doi.org/10.1242/dmm.047662.

28. Diener, C., Keller, A., and Meese, E. (2024). The miRNA–target interactions: An underestimated intricacy. Nucleic Acids Res. *52*, 1544–1557. https://doi.org/10.1093/nar/gkad1142.

29. Kern, F., Aparicio-Puerta, E., Li, Y., Fehlmann, T., Kehl, T., Wagner, V., Ray, K., Ludwig, N., Lenhof, H.-P., Meese, E., and Keller, A. (2021). miRTargetLink 2.0—interactive miRNA target gene and target pathway networks. Nucleic Acids Res. *49*, W409–W416. https://doi.org/10.1093/nar/gkab297.

30. Taylor, S.A., Schupp, J.C., Poli, S., Ayaub, E.A., Nir, N., Ahangari, F., Chu, S.G., Raby, B.A., DeIuliis, G., Januszyk, M., et al. (2020). Single-cell RNA-seq reveals ectopic and aberrant lung-resident cell populations in idiopathic pulmonary fibrosis. Sci. Adv. 6, eaba1983.

31. Kobayashi, T., Tanaka, K., Fujita, T., Umezawa, H., Amano, H., Yoshioka, K., Naito, Y., Hatano, M., Kimura, S., Tatsumi, K., and Kasuya, Y. (2015). Bidirectional role of IL-6 signal in pathogenesis of lung fibrosis. Respir. Res. *16*, 99. https://doi.org/10.1186/s12931-015-0261-z.

32. Seyhan, A.A. (2024). Trials and Tribulations of MicroRNA Therapeutics. IJMS *25*, 1469. https://doi.org/10.3390/ijms25031469.

33. Mashiko, D., Fujihara, Y., Satouh, Y., Miyata, H., Isotani, A., and Ikawa, M. (2013). Generation of mutant mice by pronuclear injection of circular plasmid expressing Cas9 and single guided RNA. Sci. Rep. *3*, 3355. https://doi.org/10.1038/srep03355.

34. Sanada, Y., Ikuta, Y., Ding, C., Yimiti, D., Kato, Y., Nakasa, T., Mizuno, S., Takahashi, S., Huang, W., Lotz, M.K., et al. (2023). miR-26a deficiency is associated with bone loss and reduced muscle strength but does not affect severity of cartilage damage in osteoarthritis. Mech. Ageing Dev. *212*, 111806. https://doi.org/10.1016/j.mad.2023.111806.

35. Karhadkar, T.R., Pilling, D., and Gomer, R.H. (2021). Serum Amyloid P inhibits single stranded RNA-induced lung inflammation, lung damage, and cytokine storm in mice. PLoS One *16*, e0245924. https://doi.org/10.1371/journal.pone.0245924.

36. Schindelin, J., Arganda-Carreras, I., Frise, E., Kaynig, V., Longair, M., Pietzsch, T., Preibisch, S., Rueden, C., Saalfeld, S., Schmid, B., et al. (2012). Fiji: an open-source platform for biological-image analysis. Nat. Methods *9*, 676–682. https://doi.org/10.1038/nmeth.2019.

37. Lianoglou, S., Garg, V., Yang, J.L., Leslie, C.S., and Mayr, C. (2013). Ubiquitously transcribed genes use alternative polyadenylation to achieve tissue-specific expression. Genes Dev. *27*, 2380–2396. https://doi.org/10.1101/gad.229328.113.

38. Ge Steven, X., Son, E.W., and Yao, R. (2018). iDEP: an integrated web application for differential expression and pathway analysis of RNA-Seq data. BMC Bioinf. *19*, 534.

39. Horimasu, Y., Ishikawa, N., Taniwaki, M., Yamaguchi, K., Hamai, K., Iwamoto, H., Ohshimo, S., Hamada, H., Hattori, N., Okada, M., et al. (2017). Gene expression profiling of idiopathic interstitial pneumonias (IIPs): identification of potential diagnostic markers and therapeutic targets. BMC Med. Genet. *18*, 88. https://doi.org/10.1186/s12881-017-0449-9.

OMTN, Volume *36*

Supplemental information

Systemic miR-26a deficiency attenuates pulmonary fibrosis via PTEN upregulation and downstream TIMP-1 suppression

Arisa Hamada, Kiyofumi Shimoji, Taku Nakashima, Kakuhiro Yamaguchi, Shinjiro Sakamoto, Yasushi Horimasu, Takeshi Masuda, Hiroshi Iwamoto, Hironobu Hamada, Yun Guo, Tomoharu Yasuda, Shigeru Miyaki, and Noboru Hattori

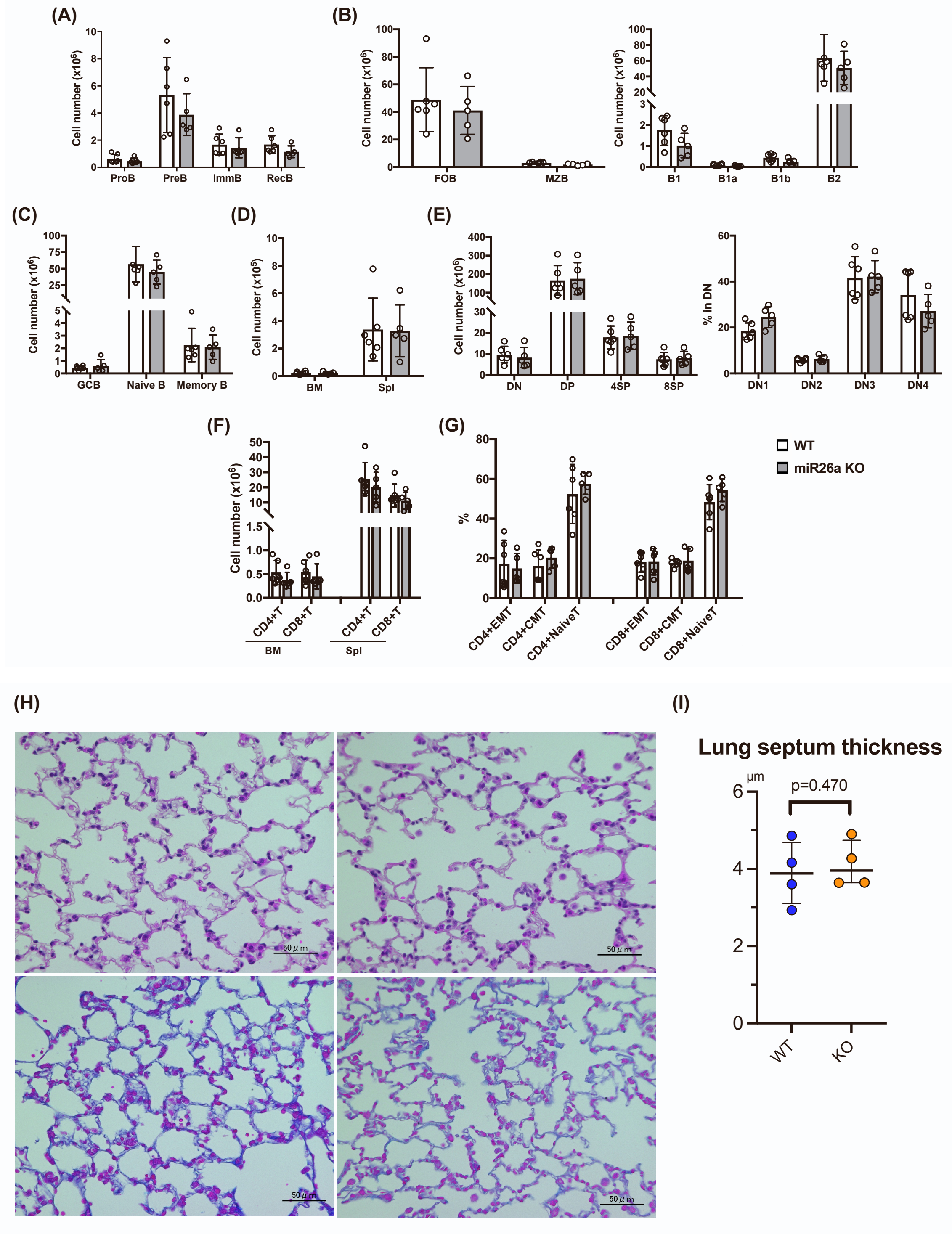

(A)
(B)
(C)
(D)
(E)
(F)
(G)
(H)
(I)
Cell number (x10⁶)
ProB
PreB
ImmB
RecB
FOB
MZB
B1
B1a
B1b
B2
GCB
Naive B
Memory B
Cell number (x10⁵)
BM
Spl
DN
DP
4SP
8SP
% in DN
DN1
DN2
DN3
DN4
CD4+T
CD8+T
%
CD4+EMT
CD4+CMT
CD4+NaiveT
CD8+EMT
CD8+CMT
CD8+NaiveT
WT
miR26a KO
50 μm
Lung septum thickness
μm
p=0.470
WT
KO

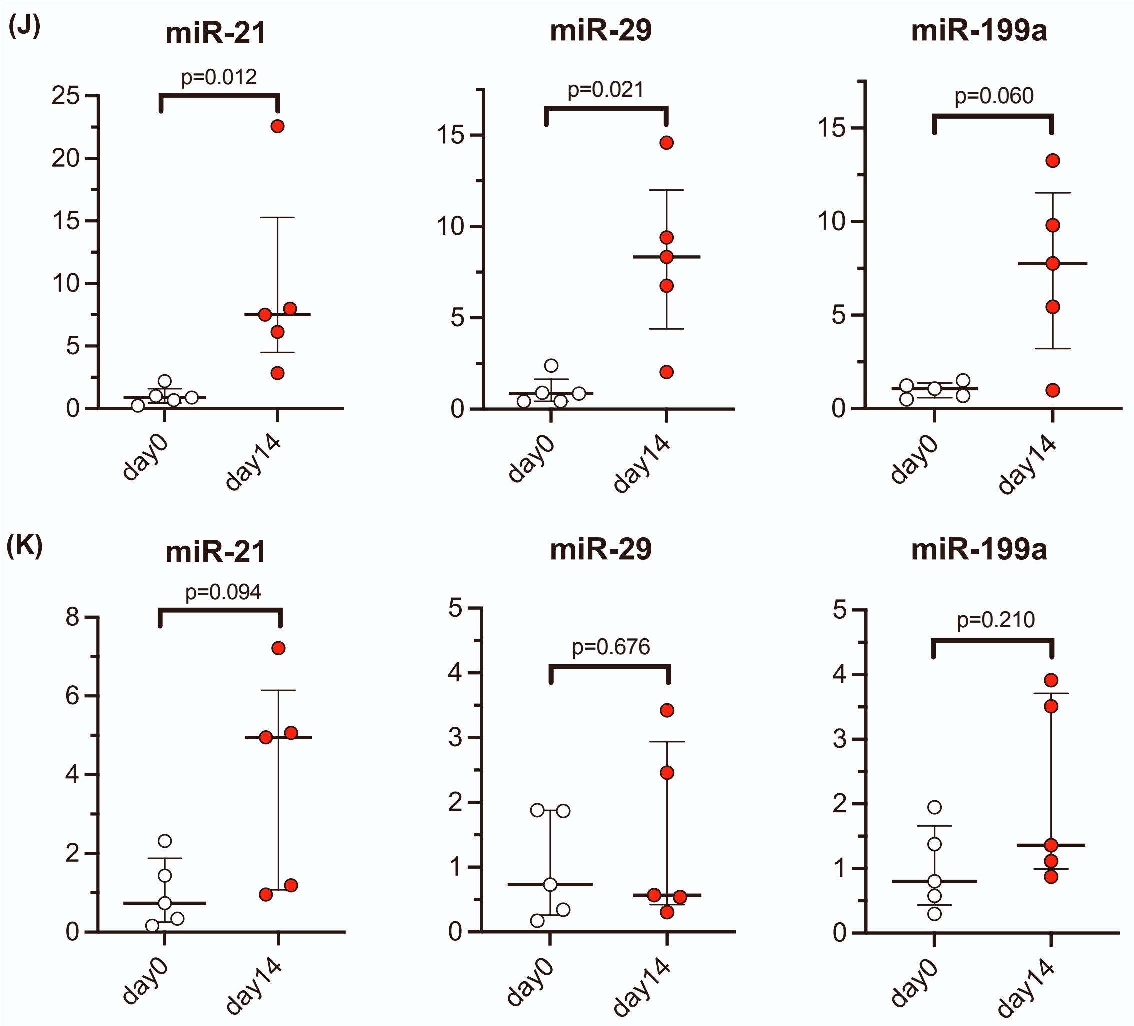

Figure S1

(A–G) Flow cytometry analysis of immune cells in bone marrow (BM), spleen (Spl), and thymus obtained from WT (*white*) and miR-26a KO (*grey*) mice (n = 5–6/group). (A) B cell development in BM. Pro-B cells, $B220^{+}CD19^{-}$; Pre-B cells, $B220^{+}CD19^{+}IgM^{-}IgD^{-}$; Immature (Imm) B cells, $B220^{+}CD19^{+}IgM^{+}IgD^{low}$; and Recirculating (Rec) B cells, $B220^{+}CD19^{+}IgM^{+}IgD^{high}$. (B and C) B1 and B2 cell development in spleen. (B) Follicular (FO) B cells, $CD19^{+}B220^{+}AA4.1^{-}CD21^{low}CD23^{high}$; Marginal zone (MZ) B cells, $CD19^{+}B220^{+}AA4.1^{-}CD21^{high}CD23^{low}$; B1 cells, $CD19^{+}B220^{low}$; B1a cells, $CD19^{+}B220^{low}CD5^{+}IgM^{high}$; B1b cells, $CD19^{+}B220^{low}CD5^{-}IgM^{high}$; and B2 cells, $CD19^{+}B220^{+}$ corresponding to FO and MZ B cells. (C) Naive B cells, $B220^{+}CD19^{+}CD38^{+}Fas^{-}$; Germinal center (GC) B cells, $B220^{+}CD19^{+}CD38^{-}Fas^{+}$; and Memory B cells, $B220^{+}CD19^{+}CD38^{+}CD73^{+}$. (D) $CD138^{+}TACI^{+}$ plasma cells in BM and spleen. (E) T cell development in thymus. DN, $CD4^{-}CD8^{-}$; DP, $CD4^{+}CD8^{+}$; 4SP,

$CD4^{+}CD8^{-}$; and 8SP, $CD4^{-}CD8^{+}$. DN cells were further fractioned to DN1 to DN4. DN1, $CD25^{-}CD44^{+}$; DN2, $CD25^{+}CD44^{+}$; DN3, $CD25^{+}CD44^{-}$; and DN4, $CD25^{-}CD44^{-}$. (F) Peripheral T cells in BM and spleen. $CD4^{+}$ T cells, $CD4^{+}CD8^{-}$; and $CD8^{+}$ T cells, $CD4^{-}CD8^{+}$. (G) Effector and memory development of $CD4^{+}$ and $CD8^{+}$ T cells in spleen. Naive T cells, $CD62L^{+}CD44^{-}$; Effector memory T cells (EMT), $CD62L^{-}CD44^{+}$; and Central memory T cells (CMT), $CD62L^{+}CD44^{+}$.

(H) Hematoxylin and eosin (HE) staining and Azan staining of WT and miR-26a KO murine lungs. Scale bars: 50 μm.

(I) The equivalent diameter of the lung septum thickness was measured and images were quantified using ImageJ2 (version 2.16.0/1.54p) (n = 4/group).

(J, K) miR-21, miR-29, and miR-199a levels in the lungs of (J) WT and (K) miR-26a KO mice before and on 14 days after BLM administration (n = 5/group).

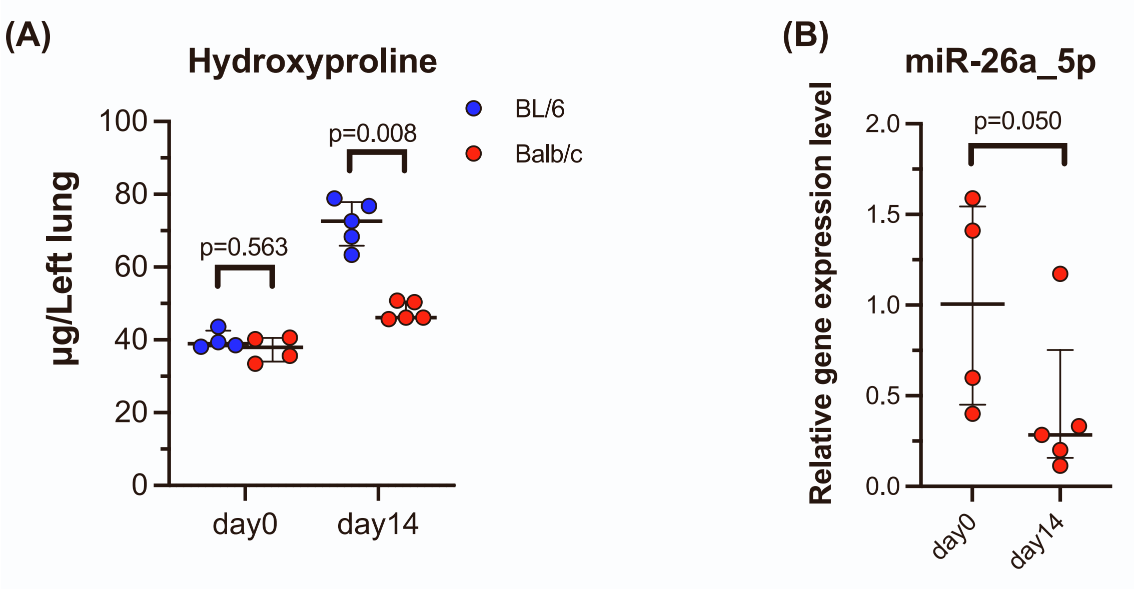

Figure S2

(A) Hydroxyproline levels in the left lung of C57BL/6J and BALB/cJ mice were measured 14 days after BLM administration (n = 4–5/group).

(B) miR-26a levels in the lungs of C57BL/6J and BALB/cJ mice before and on 14 days after BLM administration (n = 4–5/group).

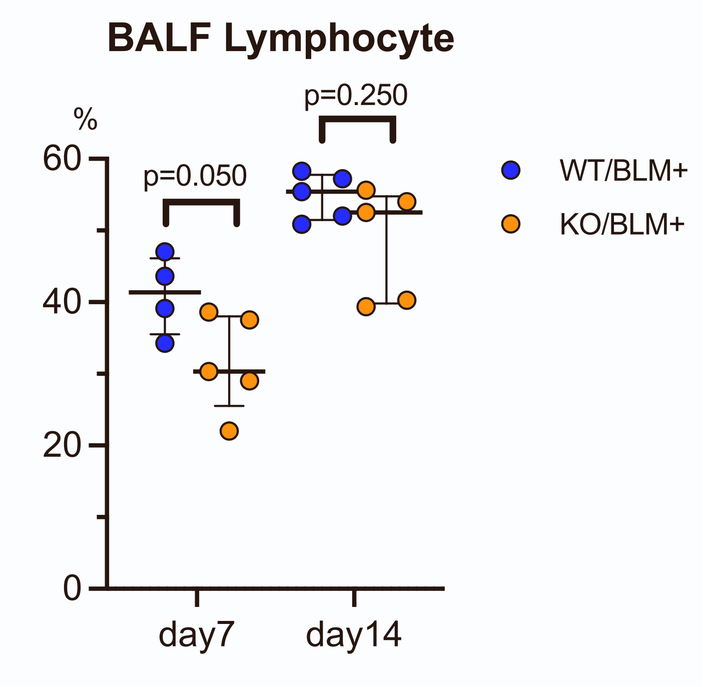

Figure S3

Lymphocytes percentages in BALF were measured 7 and 14 days after BLM administration (n = 4–5/ group).

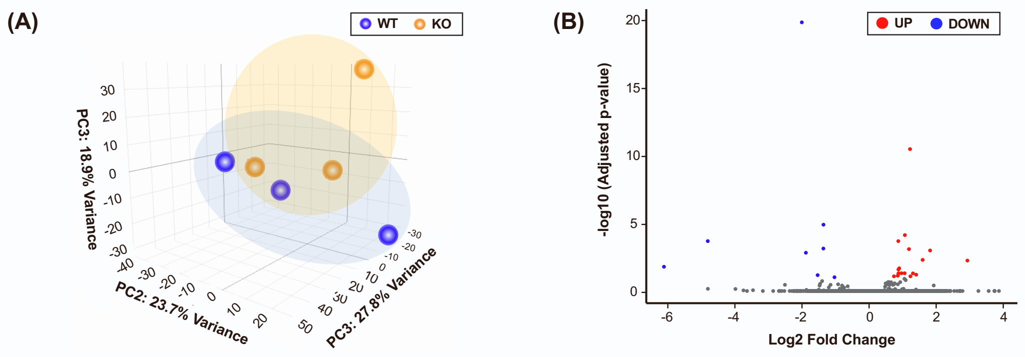

(C)

UP / DOWN	-log10 (FDR)	Fold enriched	Pathway
Upregulated	4.02	78.715	Circadian rhythm
Upregulated	1.64	24.441	Metabolism of xenobiotics by cytochrome P450
Downregulated	1.32	50.688	ECM-receptor interaction
Downregulated	1.32	44.605	Toll-like receptor signaling pathway
Downregulated	1.32	70.802	GnRH secretion
Downregulated	1.32	41.301	Protein digestion and absorption
Downregulated	1.32	32.798	Apelin signaling pathway

Figure S4

RNA sequencing of WT and miR-26a KO murine lungs was conducted on before BLM administration (n = 3/group).

(A) Each dot represents one sample subjected to principal component analysis (PCA).

(B) Volcano plot of differential gene expression between WT and miR-26a KO groups (n = 3/group) : the upregulated genes are dotted in red and downregulated in blue.

(C) Top seven enriched signaling pathways following KEGG enrichment analysis.

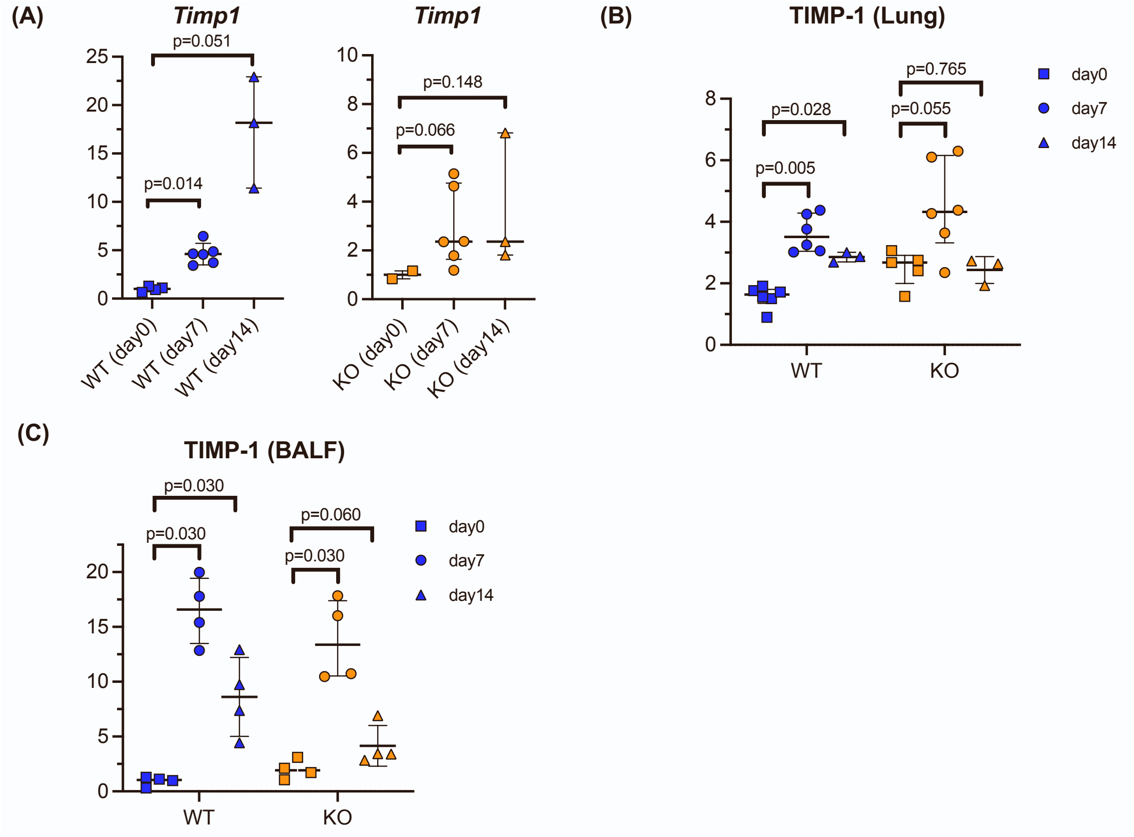

Figure S5

(A) Quantitative PCR analysis of *Timp1* mRNA expression in murine lungs before and on 7 and 14 days after BLM administration (n = 2–6/group).

(B, C) ELISA analysis of TIMP-1 protein levels in murine lungs (ng/mg Protein) and BALF (ng/mL) before and on 7 and 14 days after BLM administration (n = 3–6/group).

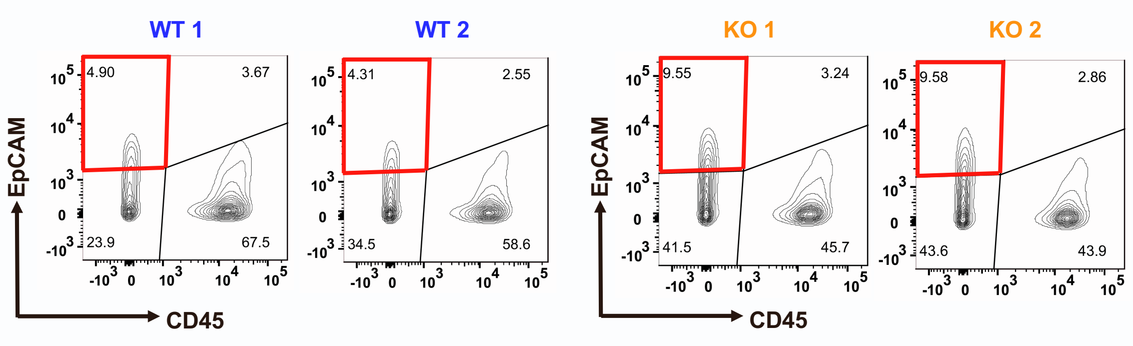

Figure S6

Flow cytometry analysis of the percentage of epithelial cells (defined as CD45⁻/EpCAM⁺) in total lung cells on 7 days after BLM administration in WT and miR-26a KO mice.

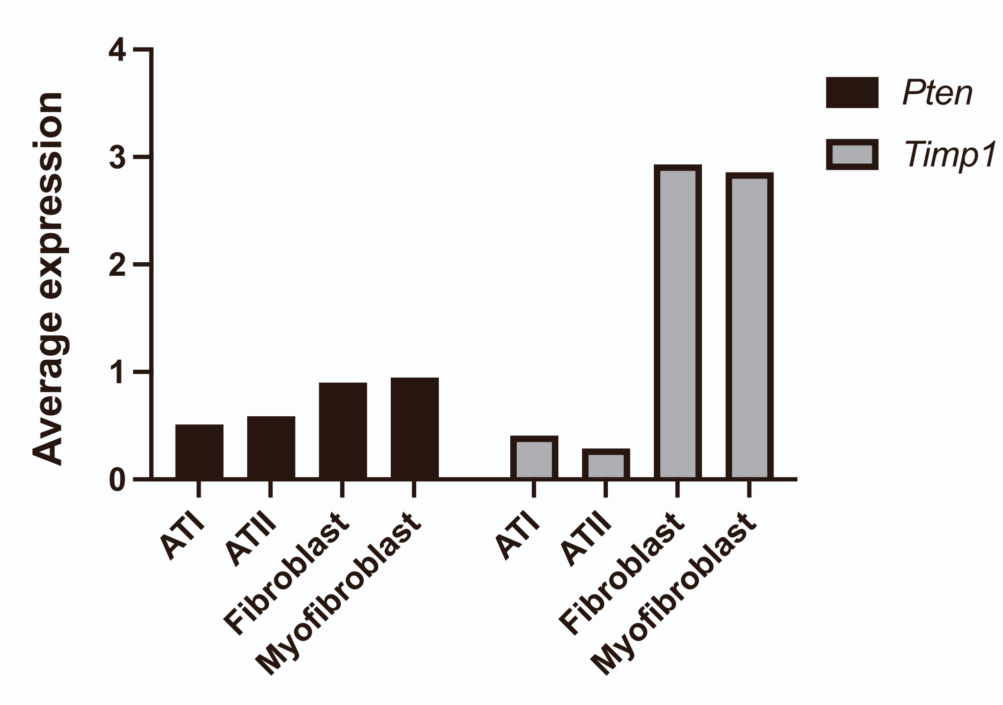

Figure S7

Supplemental figures were created with reference to the publicly available results presented on the IPF Cell Atlas website (http://ipfcellatlas.com/). No additional analyses were performed, and the original data are derived from single-cell transcriptomic data of human lung cells from patients with idiopathic pulmonary fibrosis (IPF) reported by Adams et al. (Nat Med 2020). Average expression represents the $\log_{1+x}$-transformed normalized expression values (log-normalized UMI counts) as shown in the IPF Cell Atlas.
